# Whole-Exome Sequencing, Proteome Landscape, and Immune Cell Migration Patterns in a Clinical Context of Menkes Disease

**DOI:** 10.3390/genes12050744

**Published:** 2021-05-14

**Authors:** Margarita L. Martinez-Fierro, Griselda A. Cabral-Pacheco, Idalia Garza-Veloz, Jesus Acuña-Quiñones, Laura E. Martinez-de-Villarreal, Marisol Ibarra-Ramirez, Joke Beuten, Samantha E. Sanchez-Guerrero, Laura Villarreal-Martinez, Ivan Delgado-Enciso, Iram P. Rodriguez-Sanchez, Vania Z. Zuñiga-Ramirez, Edith Cardenas-Vargas, Viktor Romero-Diaz

**Affiliations:** 1 Molecular Medicine Laboratory, Unidad Académica de Medicina Humana y C.S, Universidad Autónoma de Zacatecas, Carretera Zacatecas-Guadalajara Km.6, Ejido la Escondida, Zacatecas 98160, Mexico; gris_elda_ai91@hotmail.com (G.A.C.-P.); idaliagv@uaz.edu.mx (I.G.-V.); jesusacunaquinones@gmail.com (J.A.-Q.); vaniazhanelizr@gmail.com (V.Z.Z.-R.); 2 Departamento de Genética, Facultad de Medicina, Universidad Autónoma de Nuevo León, Monterrey 64460, Mexico; laelmar@yahoo.com.mx (L.E.M.-d.-V.); m.ibarrar25@gmail.com (M.I.-R.); 3 AiLife Diagnostics, 1920 Country Pl Pkwy Suite 100, Pearland, TX 77584, USA; yokabeuten@hotmail.com; 4 Hospital General Zacatecas “Luz González Cosío”, Servicios de Salud de Zacatecas, Zacatecas 98160, Mexico; sammelisg@gmail.com (S.E.S.-G.); hormonitazac@gmail.com (E.C.-V.); 5 Hematology Service, Hospital Universitario “Dr. José Eleuterio González”, Universidad Autónoma de Nuevo León, Monterrey 64460, Mexico; dr_lauravillarreal@hotmail.com; 6 Department of Molecular Medicine, School of Medicine, University of Colima, Colima 28040, Mexico; ivan_delgado_enciso@ucol.mx; 7 Molecular and Structural Physiology Laboratory, School of Biological Sciences, Autonomous University of Nuevo León, Monterrey 64460, Mexico; iram.rodriguezsa@uanl.edu.mx; 8 Department of Histology, Universidad Autónoma de Nuevo León, Facultad de Medicina, Monterrey 64460, Mexico; vikromero@email.com

**Keywords:** Menkes disease, silvery hair syndrome, rare disease, exome sequencing, hypopigmentary disorder

## Abstract

Menkes disease (MD) is a rare and often lethal X-linked recessive syndrome, characterized by generalized alterations in copper transport and metabolism, linked to mutations in the ATPase copper transporting α (*ATP7A*) gene. Our objective was to identify genomic alterations and circulating proteomic profiles related to MD assessing their potential roles in the clinical features of the disease. We describe the case of a male patient of 8 months of age with silvery hair, tan skin color, hypotonia, alterations in neurodevelopment, presence of seizures, and low values of plasma ceruloplasmin. Trio-whole-exome sequencing (Trio-WES) analysis, plasma proteome screening, and blood cell migration assays were carried out. Trio-WES revealed a hemizygous change c.4190C > T (p.S1397F) in exon 22 of the *ATP7A* gene. Compared with his parents and with child controls, 11 plasma proteins were upregulated and 59 downregulated in the patient. According to their biological processes, 42 (71.2%) of downregulated proteins had a participation in cellular transport. The immune system process was represented by 35 (59.3%) downregulated proteins (*p* = 9.44 × 10^−11^). Additional studies are necessary to validate these findings as hallmarks of MD.

## 1. Introduction

Menkes disease (MD), a lethal X-linked recessive syndrome that is characterized by generalized alterations in copper transport and metabolism, is linked to pathogenic variants in the ATPase copper transporting α (*ATP7A)* gene, which maps to Xq21.1 [1,2]. The overall incidence is 1 in 100,000–250,000 births, grouping MD as an ultra-rare orphan disease [3]. Genetically, loss-of-function alterations in *ATP7A*, resulting from point mutations, short indel, large deletions, and duplications, account for all the cases reported to date [1,2,4,5,6,7]. The ATP7A protein facilitates the export of copper from non-hepatic tissues and delivers copper to the secretory pathway for incorporation into copper-dependent enzymes [6]. The symptoms of MD derive from a deficiency of dietary copper absorption and copper transfer across the intestinal membrane into the circulation and from impaired reabsorption of copper in the kidney [4]. In the absence of ATP7A activity, copper accumulates in intestinal cells and the kidney, and unusually there are low levels of copper in the heart, liver, and brain [4,6]. The reduced activity of essential cuproenzymes [4,6] such as ceruloplasmin, lysyl oxidase, superoxide dismutase, tyrosinase, cytochrome C oxidase, and dopamine β-monooxygenase among others, constitutes a hallmark of MD [8,9,10]. Based on the symptoms, two forms of MD have been described: classic MD, in which the clinical features comprise neurological defects (severe mental retardation, neurodegeneration, and seizures), growth retardation, hypothermia, laxity of skin and joints, hypopigmentation, and peculiar “kinky” or “steely” hair; as well as the mildest recognized form of MD, wherein the neurological symptoms are milder, leading to a clinical picture with manifest connective tissue symptoms [9]. While patients with classic MD have life shortening (death usually occurs by 3 years of age), patients with the mild form of the disease have a longer lifespan, because neurological defects in particular are less profound [9]. Once clinical features and hair abnormalities appear, the definitive diagnosis involves low serum copper and ceruloplasmin levels, and/or high copper concentrations in cultured fibroblasts from the patient [10]. Genetic testing for mutations in *ATP7A* is desirable. 

In Mexico, there are no data of incidence of MD, and because of similarities in the clinical diagnosis to those of other hypopigmentary-related disorders, and the cost of genetic tests, it is highly probable that MD patients remain misclassified or under-diagnosed. In this study, we describe an 8-month-old male patient with silvery hair, tan skin color, neurodevelopment delays and seizures, normal levels of blood copper and low values of plasma ceruloplasmin. Trio-whole-exome sequencing (Trio-WES) analysis identified a high-impact mutation in *ATP7A.* A plasma proteomic profile was carried out to contribute to the discovery of potential biomarkers for MD.

## 2. Materials and Methods

### 2.1. Ethics

For the study, approval was given by both the Comité de Enseñanza e Investigación of the General Hospital “*Luz González Cosío*” and by the Academic Unit of Human Medicine and Health Sciences Research Committee (IDs: 0213/2019 and CI-R-0003-2020). Parents and/or patients gave informed consent and/or assent as per the Declaration of Helsinki.

### 2.2. Clinical Data and Biological Samples

**Figure 1:**
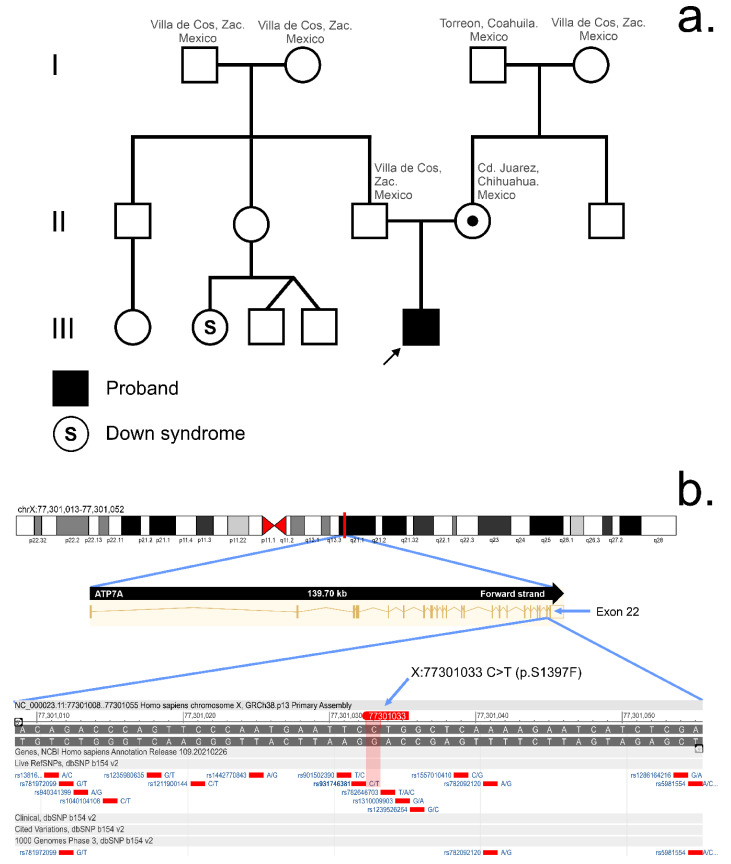
Pedigree chart and representation of the c.4190C > T variant. (**a**) The proband was born of non-consanguineous parents and he had no family history of hereditary diseases. (**b**) Representation of the location of the hemizygous c.4190C > T (*p* S1397F) variant: X: 77301033 C > T exon 22 of the ATP7A gene.

**Figure 2:**
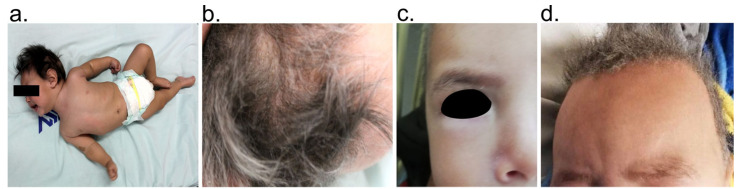
Physical features of the patient with Menkes disease. In addition to neurological alterations, features such as hypotonia (**a**), brittle hair together with alterations in the hair pigmentation (**b**) and (**d**), and hypopigmented skin areas in the face (**c**) were identified. In the second medical visitation, tanned skin (**d**) and “kinky” and “steely” scalp hair were more evident.

*Case description:* The patient, an 8-month-old male, is the first child born to non-consanguineous healthy parents (Figure 1a and Table 1). His mother is 19 years old and his father 18 years old at the time of gestation. There is no relevant family history of hereditary disease. The patient was born at 40 weeks of gestation without any complications and a weight of 4000 g. Physical examination showed Tanner 1 male genitalia, weight and length appropriate for age, head circumference of 43 cm (percentile 3rd to 15th), reactive and irritable (Figure 2a–d). Phenotypic examination showed hyperpigmentation of the skin in exposed areas, hypopigmented hair, dolichocephaly, and sparse brittle scalp hair. Facial features included high anterior hairline and large forehead, and sparse hypopigmented eyebrows, brown iris, isochoric, wide nasal bridge, long philtrum, and ears with prominent antihelix.

The patient had a full range of motion (ROM) of all joints, hypotonia without neck holding, and deep tendon reflexes NINDS Scale: 2. Global delay of neurodevelopment was also detected. The patient at the time of evaluation (eight months) had presented three episodes of generalized tonic seizures, in management and remission in treatment with levetiracetam and valproate. His skull CT scan and metabolic screening were without alterations. Abdominal ultrasonography showed his abdomen with no organomegaly or presence of masses. Abnormal curvature of spine was not observed, and his extremities had no asymmetry or muscle atrophy. At the cardiovascular level, he showed regular rate and rhythm with no presence of extra sounds or murmurs. The patient had a previous history of normal levels of plasma copper and low values of ceruloplasmin in plasma (Table 2). 

At the time of sampling (Table 3, 8 months), he had differences in the number of white blood cells compared to normal values, specifically in lymphocytes and granulocytes, and presented macrocytic anemia. In addition, he had low values of mean corpuscular volume and mean corpuscular hemoglobin, and high values of red blood cell distribution width (18.6%). There was an absence of pancytopenia, bicytopenia, and intra-cytoplasmic granules in leucocytes. Urea levels were high in all the sampling times. Both parents had no important clinical findings, and their biochemical variables were within normal values.

Throughout patient clinical follow-up (months from 2 to 20), the most frequent reason for hospitalization was for episodes of food intolerance, dehydration, diarrhea, vomit and/or fever. 

*Biological samples:* For molecular evaluations, 5 mL blood samples from the parents and the patient were collected in EDTA tubes. In addition, 2 mL of blood was collected from four healthy children with indications of surgery by trauma (with no family relationship) as control for mass spectrometry (MS) evaluation. One of them (paired with the proband by age and gender) was included as control in the cell migration analyses (see below). Samples of scalp hair from the patient and from one adult (male, 60 years old) with gray hair were also considered for the study.

### 2.3. Nucleic Acid Extraction

Genomic DNA was extracted from 100 μL of peripheral blood from the patient and his parents using the DNeasy Blood and Tissue kit (Qiagen GmbH, Hilden, Germany), following the manufacturer’s instructions. The quality and quantity of the DNA samples were determined using a NanoDrop ND-1000 spectrophotometer.

### 2.4. Whole Exome Sequencing

Next-generation sequencing (NGS): for the paired-end pre-capture library procedure, 1 μg of genomic DNA was fragmented by and ligated to multiplexing PE adapters. The adapter-ligated DNA was then PCR amplified using primers with sequencing barcodes (indices). For the target enrichment procedure, the pre-capture library was enriched by hybridizing to biotin labeled in-solution probes. For massively parallel sequencing, the post-capture library DNA was subjected to sequence analysis on the NovaSeq 6000 sequencing platform (Illumina, CA, USA). The following quality control metrics of the sequencing data are generally achieved: ≥ 94% target base covered at ≥20×, mean coverage of target bases ≥100X.

*Data analysis and interpretation (NGS pipeline version 1.1):* The output data were converted from a BCL file to a FASTQ file, and then mapped by the BWA program (reference sequence: GRCh37/Hg19). The variant calls were performed using GATK and annotations performed using an in-house developed pipeline including A-GPS^®^, I-GPS^®^, and D-GPS^®^ components. Synonymous variants, deep intronic variants not affecting splicing site, and common benign variants were excluded from interpretation unless they were previously reported as pathogenic variants. The variants were interpreted according to ACMG guidelines [18] and patient phenotypes and were classified as pathogenic (P), likely pathogenic (LP), of unknown significance (VUS), likely benign (LB), or benign (B). Trio-WES results were validated by Sanger sequencing.

### 2.5. Plasma Protein Profile Determination by Mass Spectrometry

Sample preparation and protein digestion: 200 μL of each plasma sample was thawed, and serum albumin and IgG were removed using a Pierce™ Top 2 Abundant Protein Depletion Spin Column (Thermo Fisher Scientific, Waltham, MA, USA) at room temperature (RT) according the manufacturer’s instructions. The protein pellets were dissolved in 50 mM ammonium bicarbonate. The solution was transferred into Microcon devices YM-10 (Millipore, Burlington, MA, USA). The device was centrifuged at 12,000 *g* at 4 °C for 10 min. Subsequently, 200 μL of 50 mM ammonium bicarbonate were added to the concentrate followed by centrifugation (this step was repeated once). After reducing by 10 mM DL-dithiothreitol (DTT) at 56 °C for 1 h and alkylation by 20 mM iodoacetamide (IAA) at RT in the dark for 1 h, the device was centrifuged at 12,000 *g* at 4 °C for 10 min and washed once with 50 mM ammonium bicarbonate. Ammonium bicarbonate (50 mM, 100 μL) and free trypsin were added to the protein solution at a ratio of 1:50, and the solution was incubated at 37 °C overnight. Finally, the device was centrifuged at 12,000 *g* at 4 °C for 10 min and then 100 μL of 50 mM ammonium bicarbonate added and centrifuged (this step was repeated once). The extracted peptides were lyophilized to near dryness and then resuspended in 20 μL of 0.1% formic acid before LC-MS/MS analysis.

Nano LC-MS/MS analysis: The nanoflow UPLC procedure used an Ultimate 3000 nano UHPLC system (Thermo Fisher Scientific, USA) coupled to a Q Exactive HF mass spectrometer with an ESI nanospray source. The nanocolumns used were a trapping column (PepMap C18, 100 Å, 100 μm × 2 cm, 5 μm) and an analytical column (PepMap C18, 100 Å, 75 μm × 50 cm, 2 μm). The sample volume loaded was 1 μg. Mobile phases used were A: 0.1% formic acid in water; and B: 0.1% formic acid in 80% acetonitrile. Total flow rate was 250 nL/min. LC linear gradients: from 2 to 8% buffer B over 3 min; from 8 to 20% buffer B over 53 min; from 20 to 40% buffer B over 35 min; and from 40 to 90% buffer B over 4 min. The full scan was performed from 300–1650 *m*/*z* at a resolution of 60,000 at 200 *m*/*z*; the automatic gain control target for the full scan was set to 3e6. The MS/MS scan was operated in Top 20 mode using the following settings: resolution 15,000 at 200 *m*/*z*; automatic gain control target 1 × 10^5^; maximum injection time 19 ms; normalized collision energy 28%; isolation window of 1.4 Th; charge state exclusion unassigned, 1, and >6; and dynamic exclusion 30 s.

*Data capture:* The seven raw MS files (trio and from the four healthy children controls) were analyzed and searched against the human protein database based on the species of the samples using Maxquant (1.6.2.6). The parameters were set as follows: protein modifications of carbamidomethylation (C, fixed), oxidation (M, variable); the enzyme specificity set to trypsin; the maximum missed cleavages set to 2; the precursor ion mass tolerance set to 10 ppm, and the MS/MS tolerance was 0.6 Da. 

*Plasma proteomic profiles and gene ontology analysis:* Circulating proteomic profiles under the MD genotype were determined using proteomic data from father, mother or healthy children controls as reference (denominator). Fold change cutoff was set when proteins with quantitative ratios above 2 or below 1/2 were deemed significant. Gene ontology (GO) analysis of proteomic profiles was carried out using the LAGO (Logically Accelerated GO Term Finder: https://go.princeton.edu/cgi-bin/LAGO, accessed on 13 May 2021) tool [19].

### 2.6. Peripheral Blood Cell Migration Assay

*Blood mononuclear and polymorphonuclear cell separation:* 2 mL of fresh blood samples were obtained from the patient and his parents. Additionally, 2 mL of blood from a donor healthy patient (male; seven-month-old) was obtained and used as control. Blood was carefully added to a 15 mL polypropylene tube containing 2 mL of histopaque 1119 and 1077 (Sigma-Aldrich, St. Louis, MO, USA), and centrifuged at 700 rcf for 15 min at 19 °C. After centrifugation, excess plasma was discarded and bands corresponding to the peripheral blood mononuclear cells (PBMCs) and polymorphonuclear cells (PMNCs) were recovered, placed separately in fresh polypropylene tubes and washed twice with Hanks balanced salt solution (HBSS). For erythrocyte lysis, PMN cells were resuspended in isotonic solution (4.145 g NH_4_Cl, 0.5 g KHCO_3_, 18.6 mg EDTA in 500 mL H_2_O, pH 7.3) and lysis stopped by adding wash buffer (HBSS + 10% FCS + 25 mM HEPES) and then centrifuged at 400 rcf 10 min at 19 °C. Finally, cells were resuspended in DMEM (Gibco, New York, NY, USA) cell culture medium.

*Cell migration assays:* The state of activation under the MD of PBMCs and PMNCs from the Trio and control were evaluated using a modified Boyden chamber system [20]. Immediately after counting, 1.2 × 10^5^ cells resuspended in 100 μL were added to the upper part of a transwell filter with a pore size of 3 μm (Corning, Corning, NY, USA). The bottom part of the transwell containing 500 μL of cell culture medium without serum was supplemented with chemoattractant fMLP (*N*-formyl-methionyl-phenylalanine) at a final concentration of 1 × 10^−6^ M to induce PMNC transmigration. For induction of PBMC transmigration, a cocktail of chemokine (Biorad, Hercules, CA, USA) at a final concentration of 10 pg/mL was added to the bottom of the chamber. The cells were then incubated for 90 min at 37 °C and collected. Transmigrated cells were counted by using a Neubauer chamber.

### 2.7. Statistical Analysis

Statistical analysis was performed using the GraphPad Prism Version 5.0 statistical software package. PBMC and PMNC counts were expressed as means ± standard deviation (SD). Comparisons of PBMC and PMNC counts between participants were performed using Student’s *t*-test. A *p*-value of <0.05 was considered statistically significant.

## 3. Results

### 3.1. Melanin Granule Distribution in Scalp Hair Samples

**Figure 3:**
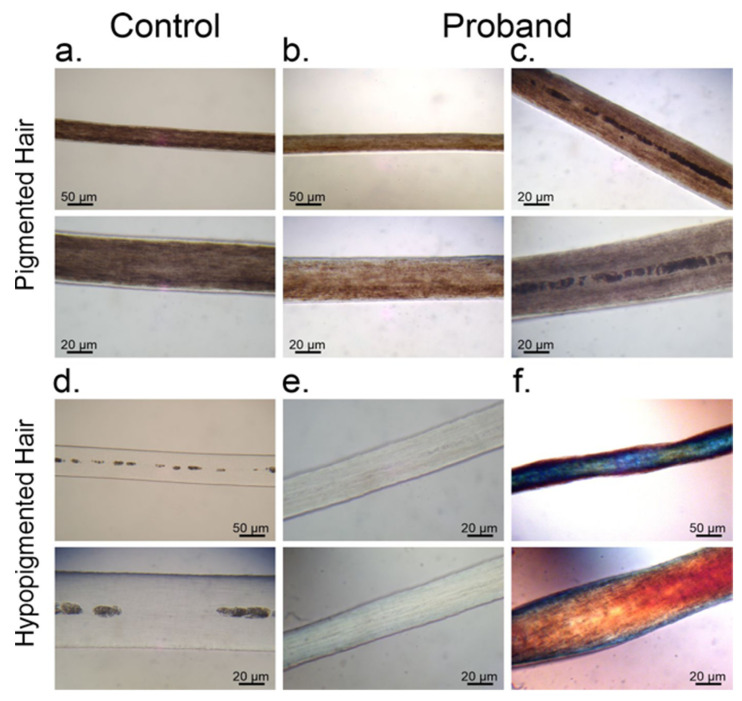
Scalp hair observations by light microscopy. Observations by light microscopy of several hair sections of adult control (**a**,**d**) and the proband (**b**,**c**,**e**,**f**). Figure shows brown (**a**) and gray hair (**d**) of the same control (male 60 years old not related with the proband). Representative colored hair sections with melanin pigment distributed homogeneously (**b**) and with central pigment accumulation (**c**) are shown. Column (**e**) shows hypopigmented hair section from the case. (**f**) Polarized light microscopic examination of the scalp hair showing pili torti structures in the hair of the proband.

Structural characteristics and melanin pigment distribution in the scalp hair were observed under light microscopy in proband (Figure 3b,c,e,f) and control (Figure 3a,d). The proband showed representative colored hair sections with melanin pigment distributed homogeneously (Figure 3b) and with central pigment accumulation (Figure 3c). The hair sections from the patient with hypopigmentation showed no Chediak Higashi syndrome (CHS) or Griscelli syndrome (GS) representative of accumulation of melanin granules in the hair (Figure 3e). Microscopic examination using polarized light showed pili torti structures in the scalp hair of the patient (Figure 3f).

### 3.2. Whole Exome Sequencing

Trio-WES showed a hemizygous c.4190C > T (p.S1397F) variant in exon 22 of the ATP7A gene (NM_000052.7; Figure 1b). The mother was heterozygous for the variant, and the father negative. This variant was classified as LP according to the ACMG (Standards and Guidelines for the Interpretation of Sequence Variants), considering at least two moderate evidences of pathogenicity and two or more supporting evidences of pathogenicity (PMID: 15981243, 28389643).

### 3.3. Mass Spectrometry Profiles

**Figure 4:**
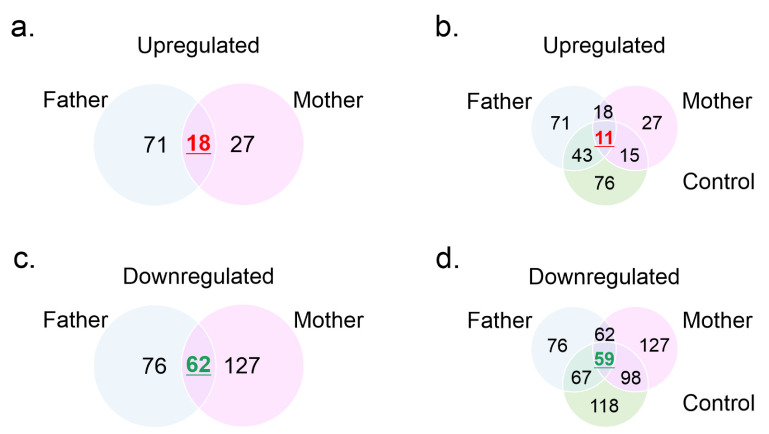
Mass spectrometry analysis profiles from human plasma samples. Plasma samples from proband, father, mother, and four healthy children controls, were analyzed by MS. Proteomic profiles were determined using proteomic data from father, mother, or healthy children controls as reference (denominator). Fold change cutoff was set when proteins with quantitative ratios above 2 or below 1/2 were deemed significant. Figure shows Venn diagrams of circulating protein upregulated (**a**,**c**) and down-regulated (**b**,**d**) between groups.

Using cultured fibroblasts from MD patients, recent evidence showed that copper-dependent trafficking was hampered because ATP7A with p.S1397F mutation was localized in the trans Golgi network (TGN) regardless of copper concentration. It was proposed that the p.S1397F mutant may affect copper uptake or copper transport directly by affecting ion-binding to the intramembrane sites [2]. To explore if this and other not described MD-abnormalities had a systemic effect, plasma MS profiles were evaluated. For this, plasma from the proband and in addition to plasma samples from his parents, four plasma samples from four children controls were included in the study as controls during the MS assays. The donors were four boys and one girl aged 1–9 years old. The results of MS analyses are displayed in Supplementary Table S1, Table 4, and Figure 4. A total of 291 plasma proteins were detected (Supplementary Table S1). Compared with his parents, the proband had 18 circulating proteins upregulated (Figure 4a) and 62 downregulated (Figure 4b). Furthermore, the patient showed 76 proteins upregulated and 118 proteins downregulated compared with healthy children controls. Considering the comparison between the three-plasma protein profiles, 11 proteins were upregulated in the patient and 59 were downregulated. Of the downregulated proteins, 27 were not detected in the patient (Figure 4c,d, and Table 4). These proteins included moesin, tubulin α-1c chain, myosin regulatory light chain 12A and H9, pleckstrin, and calreticulin, among others (Table 4). 

### 3.4. Gene Ontology Analysis

**Figure 5:**
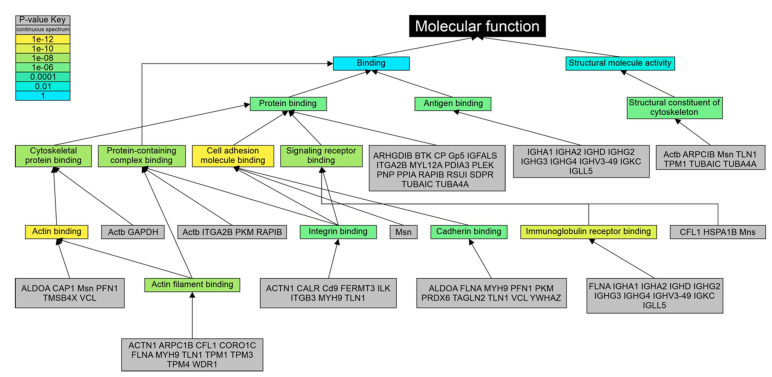
Gene ontology analysis of 59 downregulated proteins using molecular function as classificatory term. The list of 59 downregulated proteins was used as input in the LAGO tool. In the analysis, human GOA option, molecular function, *p*-value cutoff of 0.001, and Bonferroni correction, were selected. The background size shown in the Figure was of 19,680. Actin binding (*p* = 6.82 × 10^−13^), cell adhesion molecule binding (*p* = 6.08 × 10^−12^), and immunoglobulin receptor binding (*p* = 3.5 × 10^−11^) were the terms with more significant *p*-values. Protein binding (GO: 0005515) grouped all the proteins included in the analysis (*p* = 1.37 × 10^−5^).

GO analysis of the 59 common downregulated proteins revealed that 50 (84.7%) constituted a cellular component of the vesicle (GO: 0031982), 45 (76.2%) of these proteins being a component of both extracellular vesicle (GO: 1903561) and extracellular exosome (GO: 0070062; Supplementary Table S2). Classifying the downregulated proteins by biological process, 42 (71.2%) of them participated in cellular transport (GO: 0006810), with 38 (64.4%) proteins grouped in the vesicle-mediated transport (GO: 0016192); the latter GO term was the mechanism with the most significant *p*-value (*p* = 1.05 × 10^−19^; Supplementary Table S3). The immune system (GO: 0002376) was represented by 35 (59.3%) downregulated proteins (*p* = 9.44 × 10^−11^). According to their molecular function (Figure 5 and Supplementary Table S4), actin binding (GO: 0003779; *p* = 6.82 × 10^−13^), cell adhesion molecule binding (GO: 0050839; *p* = 6.08 × 10^−12^), and immunoglobulin receptor binding (GO: 0034987; *p* = 3.5 × 10^−11^) were the terms with more significant *p*-values. Protein binding (GO: 0005515) grouped all the proteins included in the analysis (*p* = 1.37 × 10^−5^). 

Supplementary Tables S5–S7 show a summary of the GO analysis for the 11 upregulated proteins. Considering the cellular component as a classifier, the most significant GO term was extracellular space (GO: 0005615; *p* = 1.8 × 10^−7^) in which all the proteins were grouped; additionally, seven proteins (HBB, LAMP2, MASP2, PRDX2, SHBG, STXBP2, TNXB) were located as part of the extracellular vesicle and/or extracellular exosome (*p* < 0.01).

### 3.5. Peripheral White Blood Cell Migration Assay

**Figure 6:**
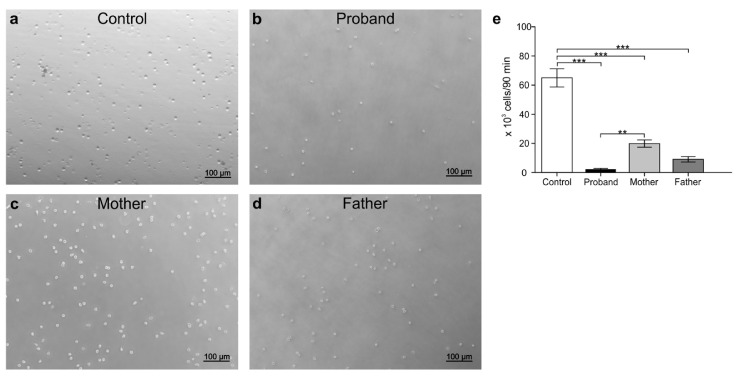
Transmigration of polymorphonuclear cells assays. Peripheral blood polymorphonuclear cells (PMNCs) were isolated from different subjects: control (**a**), proband (**b**), mother (**c**), and father (**d**). Subsequently, the cells (120,000) were added to the surface of a transwell filter (Ø = 3 μm) and stimulated with 1 μM of the chemoattractant fMLP. Afterwards, the cells were incubated at 37 °C for 90 min, the transmigrated cells subsequently counted, and the results compared using *t*-test (**e**). ** *p* < 0.01, *** *p* < 0.001. *n* = 3. Data are represented as the mean ± SD.

**Figure 7:**
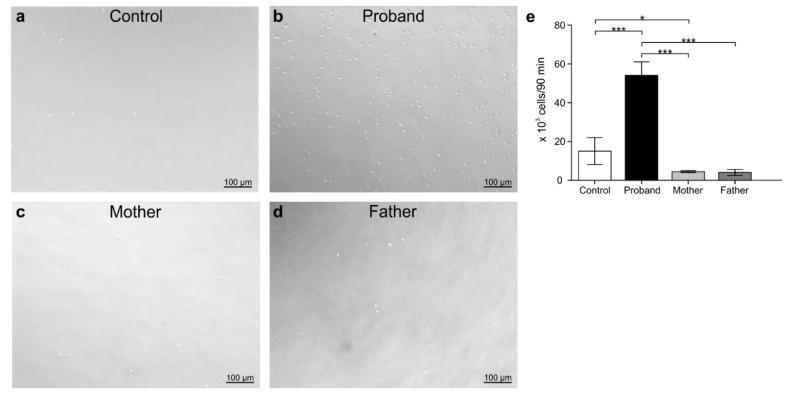
Transmigration of peripheral blood mononuclear cells assay. Peripheral blood mononuclear cells (PBMC) were isolated from different subjects: control (**a**), proband (**b**), mother (**c**), and father (**d**). Subsequently, the cells (120,000) were added to the surface of a transwell filter (Ø = 3 μm) and stimulated with 1 μM of the 10 pg/mL of chemokine cocktail (Bio-Plex Human Chemokine Assays, BioRad). Afterwards, the cells were incubated at 37 °C for 90 min and subsequently the transmigrated cells were counted, and the results compared using *t*-test (**e**). * *p* < 0.05 ** *p* ≤ 0.01, *** *p* < 0.001 (*n* = 3).

To identify the effects of MD genotype on immune cells and to determine the state of immune-cell activation under disease, transmigration assays were carried out. For this test, PBMCs and peripheral PMNCs were isolated from the blood sample from the child with MD, the father, the mother, and a non-related control (male; seven-month-old). PMNCs isolated from parents and patient, as well as from the control, were stimulated with the chemoattractant fMLP. The results are shown in Figure 6a–e. Even when the experimental conditions were the same for all treatments, the number of transmigrating PMNCs isolated from the patient was lower compared to those from the control or from his parents (*p* < 0.05). Unlike the control, PMNCs derived from the mother and father were also less migratory (Figure 6e). Contrary to those observed for PMNCs and compared to that of the healthy donor, the migration patterns of PBMCs were increased under chemokine stimulation in the patient with MD (Figure 7a–e). The numbers of transmigrating PBMCs derived from the parents were mostly similar to that from the control except in the case of the mother, where the number of PBMCs was slightly increased with respect to the control (*p* < 0.05). 

## 4. Discussion

In this study, we evaluated a male patient of 8 months old with MD, an ultra-rare orphan X-linked recessive disease, to identify genomic alterations and peripheral proteomic markers, and to contribute to an understanding of the molecular basis of the disease. In agreement with reports by other authors, in our study the progressive neuro-degeneration and connective tissue disturbances, together with the peculiar “kinky” hair, were the main indicators of MD [21]. Regarding structural characteristics and melanin pigment distribution in the scalp hair, pathognomonic pili torti structures were evident in the hair of the patient, supporting the diagnosis [22]. In addition to clinical features and hair abnormalities, it is well accepted that low serum copper and ceruloplasmin levels, and/or high copper concentrations in cultured fibroblasts from the patient are useful in the establishment of MD diagnosis [10]. In our study, blood copper was normal (60 µg/dL; normal: 20–70 µg/dl) and ceruloplasmin in plasma was low (2.5 mg/dL; normal: 5–33 mg/dL) at 2 months of age, and therefore MD diagnosis was ruled out in the first instance. The same result (normal copper levels and low ceruloplasmin values) was reproduced at 20 months of age when the diagnosis of MD had already been established. There are conditions that can raise serum copper levels, including toxicity due to copper ingestion of contaminated water or dietary supplements, anemia, liver disease, hemochromatosis, thyroid disorders (hypothyroidism or hyperthyroidism), infections, leukemia, lymphoma, and autoimmune diseases such as rheumatoid arthritis, among others [23]. In spite of this we cannot explain certainly why copper values in the proband persisted in normal ranges over time; the most frequent reasons for the patient’s admission were because he presented episodes of food intolerance, dehydration, diarrhea, vomit and/or fever. In general, within the laboratory findings, hemoglobin, hematocrit, and erythrocytes were within reference ranges but the presence of microcytosis and persistent hypochromia stands out (see Table 3). Infections of the upper respiratory tract or urinary tract were diagnosed three times. Is well known that a common hallmark of infection irrespective of the pathogenic agent (viral, bacterial, fungal) is a marked and progressive rise in serum copper. This elevation in copper is attributed to ceruloplasmin (a multicopper oxidase secreted from the liver that accounts for 95% of the copper content of the serum) [24]. Ceruloplasmin is an acute phase protein induced in response to inflammation, trauma, or infection, and its levels are also induced during infection. Since one molecule of ceruloplasmin binds six atoms of copper, even a modest increase in ceruloplasmin during infection can account for a substantial elevation in serum copper [24]. Ceruloplasmin values in our proband remained lower than reference values but it is probable that these values suffered subtle increases during infection episodes (staying below normal ranges), with consequences on the copper levels. Additional toxicity by ingestion of copper (water or food) even in low quantities, together with infection, could cause an effect of copper accumulation that could result in copper levels falsely remaining within reference ranges. These findings and considerations represent an important issue to note because they mean that such features may, in some cases, bias the diagnosis of MD.

WES has emerged as an effective diagnostic tool to uncover many rare variants (as seen in this study) in which the functional impact is unknown [25,26,27]. In our study the result of the WES for the Trio showed the patient was hemizygous for the variant c.4190C > T (p.S1397F) in the *ATP7A* gene, his mother being heterozygous and his father negative for this alteration. In Mexico, there are no MD cases reported with confirmed alterations in the gene *ATP7A*. Because of this, there is a high possibility that MD has been under-diagnosed in our country, and actually there are no treatments available for these patients. The *ATP7A* gene contains 23 exons distributed over approximately 140 kb of genomic DNA and encodes a protein of 1500 amino acids [3,28]. The residue change from serine to phenylalanine at position 1397 is located in transmembrane domain 8 (TMD8) of ATP7A [4]. Supplementary Table S8 summarizes the main known pathogenic mutations of the *ATP7A* gene and their association with MD. Missense mutations both in exon 22 (TMD7) and TMD8 have also been associated with MD in different populations and is thought to affect conserved residues in these domains [5,6,7,29,30,31]. There is only one case of MD reported in the world with p.S1397F mutation on *ATP7A* [4]. This was a German case with a phenotype of classical MD (information obtained by direct communication with the corresponding author). The case with this mutation has been mentioned in two additional studies by the same authors [2,9]. The most recent of these studies evaluated a library of cultured fibroblasts from 36 patients with MD and reported that the p.S1397F mutant prevents copper-induced trafficking from TGN to cytoplasmic vesicles or plasma membrane [2]. Copper-dependent trafficking was hampered because ATP7A with p.S1397F mutation was localized in the TGN regardless of copper concentration. It was proposed that the p.S1397F mutant may affect copper uptake or copper transport directly by affecting ion-binding to the intramembrane sites [2]. 

Normal copper homeostasis is essential for human growth and development [32]. The clinical consequences of copper deficiency include neurological effects, blood vessel abnormalities and impaired immune response [8,32]. Copper overload associated with mutations is related to morphological and metabolic changes in tissues [32], leading to the production of damaging free radicals and subsequent DNA cleavage [8] and, if untreated, eventual death [32]. In our study, the systemic effects of tissue abnormalities related to the phenotypical MD were evident at the circulation level, where compared to his parents and healthy children, 59 plasma proteins were downregulated and 11 upregulated in the child with MD. Interestingly, of the 59 downregulated proteins, 27 were not detected in the proband and therefore their absence has a strong potential as a biomarker for MD. These proteins included moesin, tubulin α-1c chain, myosin regulatory light chain 12A and H9, pleckstrin, integrin β-3, calreticulin, tropomyosin α-3 chain, adenylyl cyclase-associated protein 1, and coronin-1C, among others. Most of the proteins with abnormal regulation participate in cell–cell recognition, immune response, immunoglobulin receptor binding, cell transport, and cell movement, but they had not previously been evaluated in the context of MD; however, together these results reinforce the understanding of the participation of those processes in the pathophysiology of MD. Is important to note that women who are heterozygous for a pathogenic variant of *ATP7A* are typically asymptomatic, in some cases due to a favorably skewed X chromosome inactivation (PMID) [33]. In our study, the mother of proband was carrier with not apparent MD signs. However, although the X-inactivation pattern correlates somewhat with presence or absence of MD signs in female carriers, it does not sufficiently explain the observed phenotypes in all of them (PMID) [33]. Our study did not explore if the carrier status of the mother of proband had consequences in the systemic proteomic profiles obtained. Therefore, the evaluation and validation of the previously mentioned proteins in future MD studies would lead to the establishment of one or more legitimate markers for the disease. 

In our study, the effects of MD on immune cells and the state of immune cell activation under the disease were determined by using transmigration assays. Our results showed a remarkable defect in the transmigration capacity of neutrophil cells isolated from the parents, which was aggravated in the patient with MD. Neutrophil function abnormalities, including defects in transmigration capacity, can account for immune deficiencies as seen in CHS or GS-2 syndromes [34,35]. Even when cell adhesion was not evaluated and molecular analysis for the pathways involved remains to be elucidated, it is possible to speculate that migratory defects observed in the proband may be related with a failure to regulate the actin cytoskeleton and vesicle trafficking, unifying this cell component as a key player in MD as observed in neutrophil-related diseases [34,35]. Contrary to that observed for PMNCs, under chemokine stimulation, the migration of monocytes was increased in the patient with MD. Together with neutrophil, the role of monocytes in innate immune response against infections is well known [36]. The presence of frequent episodes of infection in the proband made a state of pre-activation of PBMCs highly probable, which could explain the increased migration phenotype of monocytes observed in our study. To date, there are no similar studies reporting this. However, immune response was evaluated by Nakagawa and collaborators in a macular mutant mouse (a model for Menkes kinky-hair disease) [37]. The authors demonstrated that the responses of lymph node cells, spleen cells (among other subpopulations of immune cells), and/or antibody production against sheep red blood cells, were decreased or suppressed in ml/y (hemizygous) mice, when compared to cells from +/y (control) mice [37]. Although the Nakagawa study and ours are not comparable (because the authors used a mutant murine model of the Menkes kinky hair disease, characterized by a copper deficiency in serum and in our study the patient presented normal levels of copper), both kinds of results support the hypothesis that abnormalities in immune response are associated with the MD phenotype. Is important to note that immunity changes throughout one’s life and the efficacy of the immune system deteriorates with increasing age [38]. In our migration assays, the PBMC and PMNC migration patterns of the proband were compared with that obtained from his parents and a control child (one month of age younger than the control). In spite of the cell migration patterns obtained in our study, these were consistent between assays and the cell transmigration capacity in the control was not decreased, so additional studies will be needed to evaluate if there was an age-related effect that may have introduced some bias on the observed cell migration results. 

To the best of our knowledge, our study is the first to evaluate whole exome sequencing to identify the causal mutation of MD in a Mexican patient, as well as the first study to analyze his proteomic profile, to propose a set of biomarkers with strong potential value for diagnosis and/or as biological targets, and the first to describe abnormal migration patterns of immune cells associated with MD. This study also represents the first report in America and the second in the world of an MD case with the missense mutation p.S1397F in the *ATP7A* gene. Despite additional studies being necessary to support our results as indicating hallmarks of MD, our findings contribute to a better understanding of the molecular basis of MD and refines the phenotype related to *ATP7A* mutations. 

## Figures and Tables

**Table 1:** General characteristics of the patient.

Characteristic	Finding
Age (months)	8
Sex	Male
Consanguinity	Absent
Ethnicity	Mexican
Fever	Absent
Abdominal distension	Absent
Anasarca	Absent
Paleness of body	Present
Excessively fair skin	Absent
History of similar complaints	Absent
Developmental delay	
1. Cephalic support 2. Sedestation	Absent Absent
Silvery grey hair	Present

**Table 2:** Age-adjusted copper and ceruloplasmin values.

Analyte	Reference Values		Proband Age (This Study)	
	0–6 Months	6–24 Months	2 Months	20 Months
Copper [11,12,13,14]	20–70 µg/dL	72–178 µg/dL	60 µg/dL	90 µg/dL
Ceruloplasmin [13,15,16,17]	5–33 mg/dL	26–55 mg/dL	2.5 mg/dL	16.1 mg/L

**Table 3:** Laboratory findings and monitoring of the clinical course of the disease.

Finding	Reference Range	2 Months	8 Months ^†^	14 Months	16 Months	20 Months
1. Hematic Biometry						
Leucocytes (10^3^/µL)	6–14	10.4	6.0	19.8	15.8	11.3
Lymphocytes (%)	50–60	66.8	83.1	51.7	44.5	42.7
Monocytes (%)	2–11	0	2.7	15.0	1	9
Granulocytes (%)	33	20.4	13.4	32.2	48	48.4
Lymphocyte number (10^3^/µL)	1–9	6.9	4.96	10.2	7.1	4.8
Monocyte number (10^3^/µL)	<1.0	0	0.16	2.97	0.15	1.01
Granulocyte number (10^3^/µL)	1.5–8.5	2.2	0.80	6.39	7.74	5.5
Red cell blood (10^6^/µL)	3.8–5.4	4.6	5.9	5.8	5.6	6.3
Hemoglobin (g/dL)	10.5–14	10.9	12.1	12.0	11.6	13.1
Hematocrit (%)	32–42	32.8	38.2	39.7	36.8	43.2
Mean corpuscular volume (fl)	72–88	71.5	64.3	68.3	65.2	66.8
Mean corpuscular hemoglobin (pg)	24–30	23.7	20.4	20.7	20.6	20.7
Red blood cell distribution width (%)	11.5–16	14.3	18.6	19.2	16.1	16.7
Platelets (10^3^/µL)	150–450	789	220.0	177.0	435	657
Mean platelet volume (Fl)	6–9.5	9.4	*	*	10.1	9.1
2. Blood Chemistry Test						
Glucose (mg/dL)	70–110	68	104	72	102	86
Creatinine (mg/dL)	0.12–1.06	0.33	0.36	0.37	0.21	0.39
Urea (mg/dL)	2.0–6.2	20.9	17.3	39.3	1.7	22.8
Blood urea nitrogen (mg/dL)	8–28	10	8.1	18.0	*	17
3. Chemistry Urine Test						
pH	5.5–7.0	7.0	6.0	7.0	**8.0**	6.0
Density (mg/dL)	1.005–1.010	1.003	1.029	1.011	1.020	1.027
Protein (mg/dL)	negative	negative	25	negative	negative	25
Blood (Ery/mL)	negative	negative	negative	negative	negative	10
Ketones (mg/dL)	negative	negative	negative	negative	5	15
Glucose (mg/dL)	negative	negative	negative	negative	negative	negative
Bilirubin (mg/dL)	negative	negative	negative	negative	negative	negative
Urobilinogen (mg/dL)	<2.0	normal	normal	normal	normal	normal
Nitrites (mg/dL)	negative	negative	negative	negative	negative	negative
4. Spleno-hepathomegaly (presence/absence)	–	absence	absence	absence	absence	absence
5. Pancytopenia (yes/not)	–	not	not	not	not	not
6. Bicytopenia (anemia + thrombocytopenia)	–	not	not	not	not	not
7. Intra-citoplasmic granules (leucocytes)	–	not	not	not	not	not
8. Immune dysfunction (B o T cell)	–	yes	yes	yes	yes	yes
9. Hemophagocytosis	–	not	not	not	not	not
10. Triglycerides increased	–	*	*	*	not	*
11. Fibrinogen decreased	–	*	*	not	*	*
12. Decreased albumin	–	not	not	not	not	not
13. Neurological manifestation						
Hypotonia	–	yes	yes	yes	yes	yes
Seizures	–	yes	not	not	not	not
14. Treatment (supportive) **	–	yes	yes	yes	yes	yes

^†^ Moment in which the blood samples for whole exome sequencing, mass spectrometry, and transmigration assays were taken. * Not evaluated. ** Physical rehabilitation. Abnormal values are highlighted in bold.

**Table 4:** Plasma proteins with differences between groups.

Gene Name	Protein Name	Ratio		
		Proband/Control	Proband/Father	Proband/Mother
**MSN**	Moesin	*	*	*
IGHG4	Ig γ-4 chain C region	*	*	*
IGHD	Ig delta chain C region	*	*	*
IGHA2	Ig α-2 chain C region	*	*	*
PRDX6	Peroxiredoxin-6	*	*	*
TUBA1C	Tubulin α-1C chain	*	*	*
MYL12A	Myosin regulatory light chain 12A	*	*	*
MYH9	Myosin regulatory light chain H9	*	*	*
TUBA4A	Tubulin α-4A chain	*	*	*
PNP	Purine nucleoside phosphorylase	*	*	*
TPM3	Tropomyosin α-3 chain	*	*	*
RAP1B	Ras-related protein Rap-1b	*	*	*
ARPC1B	Actin-related protein 2/3 complex subunit 1B	*	*	*
ILK	Integrin-linked protein kinase	*	*	*
WDR1	WD repeat-containing protein 1	*	*	*
SDPR	Serum deprivation-response protein	*	*	*
ITGB3	Integrin β-3	*	*	*
PLEK	Pleckstrin	*	*	*
TPM1	Tropomyosin α-1 chain	*	*	*
HSPA1B	Heat shock 70 kDa protein 1B	*	*	*
CALR	Calreticulin	*	*	*
PDIA3	Protein disulfide-isomerase A3	*	*	*
BTK	Tyrosine-protein kinase BTK	*	*	*
GP5	Platelet glycoprotein V	*	*	*
ARHGDIB	Rho GDP-dissociation inhibitor 2	*	*	*
CAP1	Adenylyl cyclase-associated protein 1	*	*	*
CORO1C	Coronin-1C	*	*	*
ACTN1	α-actinin-1	0.01	0.02	0.00
IGHG2	Ig γ-2 chain C region	0.01	0.02	0.12
YWHAZ	14–3-3 protein zeta/delta	0.02	0.02	0.005
FLNA	Filamin-A	0.02	0.24	0.005
RSU1	Ras suppressor protein 1	0.02	0.02	0.003
ITGA2B	Integrin α-IIb	0.03	0.03	0.004
FERMT3	Fermitin family homolog 3	0.03	0.01	0.004
IGHG3	Ig γ-3 chain C region	0.03	0.04	0.13
TPM4	Tropomyosin α-4 chain	0.03	0.03	0.01
TLN1	Talin-1	0.04	0.24	0.01
IGHA1	Ig α-1 chain C region	0.04	0.09	0.28
calm1a	Calmodulin	0.06	0.03	0.02
CFL1	Cofilin-1	0.06	0.05	0.01
VCL	Vinculin	0.06	0.10	0.01
IGKC	Ig kappa chain C region	0.08	0.08	0.20
TMSB4X	Thymosin β-4	0.08	0.04	0.09
TAGLN2	Transgelin-2	0.09	0.05	0.01
ACTB	Actin, cytoplasmic 1	0.09	0.13	0.01
IGKV3D-7	Ig kappa chain V-III region POM	0.13	0.27	0.30
IGLL5	Immunoglobulin lambda-like polypeptide 5	0.13	0.12	0.35
PFN1	Profilin-1	0.14	0.12	0.02
CP	Ceruloplasmin	0.15	0.11	0.15
IGKV3D-11	Ig kappa chain V-III region VG	0.16	0.41	0.46
-	-	0.16	0.13	0.35
IGHV3–49	-	0.16	0.33	0.46
CD9	CD9 antigen	0.19	0.12	0.02
F13A1	Coagulation factor XIII A chain	0.22	0.17	0.03
PKM	Pyruvate kinase PKM	0.23	0.30	0.03
PPIA	Peptidyl-prolyl cis-trans isomerase A	0.25	0.34	0.04
GAPDH	Glyceraldehyde-3-phosphate dehydrogenase	0.39	0.19	0.04
IGFALS	Insulin-like growth factor-binding protein complex acid labile subunit	0.46	0.25	0.24
ALDOA	Fructose-bisphosphate aldolase A	0.48	0.42	0.04
TNXB	Tenascin-X putative tenascin-XA	1.73	**	**
HGFAC	Hepatocyte growth factor activator	2.07	6.00	3.18
PI16	Peptidase inhibitor 16	2.38	3.23	2.51
HBB	Hemoglobin subunit β	2.62	2.33	3.99
MST1	Hepatocyte growth factor-like protein	3.02	7.70	2.63
SHBG	Sex hormone-binding globulin	3.87	**	2.53
LAMP2	Lysosome-associated membrane glycoprotein 2	4.37	2.60	5.71
MASP2	Mannan-binding lectin serine protease 2	6.28	**	6.63
PRDX2	Peroxiredoxin-2	7.54	7.48	2.88
HBG2	Hemoglobin subunit γ-2	12.28	5.58	15.35
STXBP2	Syntaxin-binding protein 2	**	**	163.70

Normalized LFQ protein intensity values were used to calculate the ratios using father, mother, or healthy children’s values as controls (denominator). Ratio cutoff values above 2 (upregulated) or below 0.5 (downregulated) were considered as significant. * Not detected in proband. ** Not detected in the reference (father, mother, or healthy children controls).

## Data Availability

Data are contained within the Supplementary Material. The data presented in this study are available in Table S1–S8 DAS needed.

## Supplementary Materials

The following are available online at https://www.mdpi.com/article/10.3390/genes12050744/s1, Table S1: UPLC-ESI-MS/MS analysis of plasma from Child with MD, his parents and healthy children controls; Table S2: Result table of gene ontology analysis for downregulated proteins considering the cellular component as classificatory; Table S3: Result table of gene ontology analysis for downregulated proteins considering the biological process as classificatory; Table S4: Result table of gene ontology analysis for downregulated proteins considering the molecular function as classificatory; Table S5: Result table of gene ontology analysis for upregulated proteins considering the cellular component as classificatory; Table S6: Result table of gene ontology analysis for upregulated proteins considering the biological process as classificatory; Table S7: Result table of gene ontology analysis for upregulated proteins considering the molecular function as classifier; Table S8. Summary of mutations in ATP7A gene.
