# Supplementary material for: Whole-Exome Sequencing, Proteome Landscape, and Immune Cell Migration Patterns in a Clinical Context of Menkes Disease
Source: Genes (Basel). 2021 May 14;12(5):744. doi: 10.3390/genes12050744 (PMC8156642; doi:10.3390/genes12050744)
Supplement: Supplementary file 1 [file genes-12-00744-s001.zip › genes-1168754-supplementary.pdf]

genes

MDPI

-Supplementary Material-

Whole-exome sequencing, proteome landscape, and immune cell migration patterns in a clinical context of Menkes disease

Margarita L Martinez-Fierro [1,*], Griselda A Cabral-Pacheco [1], Idalia Garza-Veloz [1], Jesus Acuña-Quiñones [1], Laura E. Martinez-de-Villarreal [2], Marisol Ibarra-Ramirez [2], Joke Beuten [3], Samantha E Sanchez-Guerrero [4], Laura Villarreal-Martinez [5], Ivan Delgado-Enciso [6], Iram P Rodriguez-Sanchez [7], Vania Z Zuñiga-Ramirez [1], Edith Cardenas-Vargas [2], and Viktor Romero-Diaz [8]

1 Molecular Medicine Laboratory, Unidad Académica de Medicina Humana y C.S, Universidad Autónoma de Zacatecas, Carretera Zacatecas-Guadalajara Km.6. Ejido la Escondida, 98160, Zacatecas, Mexico; margaritamf@uaz.edu.mx (M.L.M.F); gris_elda_ai91@hotmail.com (G.A.C.P); idaliagv@uaz.edu.mx (I.G.V); jesusacunaquinones@gmail.com (J.A.Q).

2 Departamento de Genética. Universidad Autónoma de Nuevo León. Facultad de Medicina, 64460, Mexico; laelmar@yahoo.com.mx (L.E.M.V); m.ibarrar25@gmail.com (M.I.R).

3 AiLife Diagnostics, 1920 Country Pl Pkwy Suite 100 Pearland (TX); yokabeuten@hotmail.com (J.B)

4 Hospital General Zacatecas *"Luz González Cosío"*, Servicios de Salud de Zacatecas, 98160, Zacatecas, México; sammelisg@gmail.com (S.E.S.G); hormonitazac@gmail.com (E.C.V).

5 Hematology Service, Hospital Universitario *"Dr. José Eleuterio González"*, Universidad Autónoma de Nuevo León, Monterrey, 64460, Mexico; dr_lauravillarreal@hotmail.com (L.V.M)

6 Department of Molecular Medicine, School of Medicine, University of Colima, Colima, 28040, México; ivan_delgado_enciso@ucol.mx (I.D.E)

7 Molecular and Structural Physiology Laboratory, School of Biological Sciences, Autonomous University of Nuevo León, Monterrey, Nuevo León, 64460, México; iram.rodriguezsa@uanl.edu.mx (I.P.R.S)

8 Department of Histology. Universidad Autónoma de Nuevo León. Facultad de Medicina, 64460, Mexico; vikromero@email.com (V.R.D)

*Correspondence: margaritamf@uaz.edu.mx; Tel.: +52 (492) 9256690 Ext/Fax: 4511 (M.L.M.F)

Supplementary Table S1 - UPLC-ESI-MS/MS analysis of plasma from Child with MD, his parents and healthy children control

Order	Protein ID	Proband: Father ratio		Proband: Mother ratio		Proband: Control ratio	
1	P26038	*	DOWN	*	DOWN	*	DOWN
2	P01861	*	DOWN	*	DOWN	*	DOWN
3	P0DOX3	*	DOWN	*	DOWN	*	DOWN
4	P0DOX2	*	DOWN	*	DOWN	*	DOWN
5	P30041	*	DOWN	*	DOWN	*	DOWN
6	Q9BQE3	*	DOWN	*	DOWN	*	DOWN
7	P19105	*	DOWN	*	DOWN	*	DOWN
8	P35579	*	DOWN	*	DOWN	*	DOWN
9	P68366	*	DOWN	*	DOWN	*	DOWN
10	P00491	*	DOWN	*	DOWN	*	DOWN
11	P06753	*	DOWN	*	DOWN	*	DOWN
12	P61224	*	DOWN	*	DOWN	*	DOWN
13	O15143	*	DOWN	*	DOWN	*	DOWN
14	Q13418	*	DOWN	*	DOWN	*	DOWN
15	O75083	*	DOWN	*	DOWN	*	DOWN
16	O95810	*	DOWN	*	DOWN	*	DOWN
17	P05106	*	DOWN	*	DOWN	*	DOWN
18	P08567	*	DOWN	*	DOWN	*	DOWN
19	P09493	*	DOWN	*	DOWN	*	DOWN
20	P0DMV9	*	DOWN	*	DOWN	*	DOWN
21	P27797	*	DOWN	*	DOWN	*	DOWN
22	P30101	*	DOWN	*	DOWN	*	DOWN
23	Q06187	*	DOWN	*	DOWN	*	DOWN
24	P40197	*	DOWN	*	DOWN	*	DOWN
25	P52566	*	DOWN	*	DOWN	*	DOWN
26	Q01518	*	DOWN	*	DOWN	*	DOWN
27	Q9ULV4	*	DOWN	*	DOWN	*	DOWN
28	Q8NBL1	*	DOWN	-	-	*	DOWN
29	P00558	-	-	*	DOWN	*	DOWN
30	P07900	-	-	*	DOWN	*	DOWN
31	P0DJI9	-	-	*	DOWN	*	DOWN
32	P11142	-	-	*	DOWN	*	DOWN
33	P60174	-	-	*	DOWN	*	DOWN
34	P31146	-	-	*	DOWN	*	DOWN
35	Q99497	-	-	*	DOWN	*	DOWN
36	P06310	-	-	*	DOWN	*	DOWN
37	P60660	-	-	*	DOWN	*	DOWN
38	P07437	-	-	*	DOWN	*	DOWN
39	O00151	-	-	*	DOWN	*	DOWN
40	O00299	-	-	*	DOWN	*	DOWN
41	P01594	-	-	*	DOWN	*	DOWN
42	P01599	-	-	*	DOWN	*	DOWN
43	P04179	-	-	*	DOWN	*	DOWN
44	P04792	-	-	*	DOWN	*	DOWN
45	P07237	-	-	*	DOWN	*	DOWN
46	P48059	-	-	*	DOWN	*	DOWN
47	P50552	-	-	*	DOWN	*	DOWN
48	P52565	-	-	*	DOWN	*	DOWN
49	P55209	-	-	*	DOWN	*	DOWN
50	P61158	-	-	*	DOWN	*	DOWN
51	P61160	-	-	*	DOWN	*	DOWN
52	P61981	-	-	*	DOWN	*	DOWN
53	Q04917	-	-	*	DOWN	*	DOWN
54	Q05682	-	-	*	DOWN	*	DOWN

55	Q15942	-	-	*	DOWN	*	DOWN
56	Q3ZCW2	-	-	*	DOWN	*	DOWN
57	Q9H4B7	-	-	*	DOWN	*	DOWN
58	Q9HBI1	-	-	*	DOWN	*	DOWN
59	P02533	-	-	-	-	*	DOWN
60	P02741	-	-	-	-	*	DOWN
61	A0A075B6K4	-	-	-	-	*	DOWN
62	P01706	-	-	-	-	*	DOWN
63	P12814	0.023	DOWN	0.001	DOWN	0.012	DOWN
64	P01859	0.022	DOWN	0.117	DOWN	0.015	DOWN
65	P63104	0.021	DOWN	0.005	DOWN	0.019	DOWN
66	P0DJI8	**	UP	0.633	[OT]	0.021	DOWN
67	P21333	0.242	DOWN	0.005	DOWN	0.022	DOWN
68	Q15404	0.020	DOWN	0.003	DOWN	0.024	DOWN
69	P08514	0.033	DOWN	0.004	DOWN	0.025	DOWN
70	Q86UX7	0.012	DOWN	0.004	DOWN	0.026	DOWN
71	P01860	0.041	DOWN	0.130	DOWN	0.028	DOWN
72	P67936	0.033	DOWN	0.009	DOWN	0.034	DOWN
73	Q9Y490	0.238	DOWN	0.005	DOWN	0.035	DOWN
74	P01876	0.092	DOWN	0.285	DOWN	0.044	DOWN
75	P0DOX5	0.057	DOWN	0.508	[OT]	0.045	DOWN
76	P0DP25	0.027	DOWN	0.022	DOWN	0.056	DOWN
77	P23528	0.055	DOWN	0.013	DOWN	0.062	DOWN
78	P18206	0.102	DOWN	0.008	DOWN	0.064	DOWN
79	Q9C0B1	0.043	DOWN	**	UP	0.068	DOWN
80	P01834	0.079	DOWN	0.200	DOWN	0.079	DOWN
81	P62328	0.041	DOWN	0.091	DOWN	0.082	DOWN
82	P37802	0.055	DOWN	0.014	DOWN	0.090	DOWN
83	P60709	0.134	DOWN	0.014	DOWN	0.091	DOWN
84	P30453	**	UP	0.024	DOWN	0.093	DOWN
85	P01624	0.273	DOWN	0.296	DOWN	0.129	DOWN
86	P0DOX8	0.121	DOWN	0.345	DOWN	0.130	DOWN
87	P07737	0.123	DOWN	0.020	DOWN	0.137	DOWN
88	P17936	0.237	DOWN	0.628	[OT]	0.149	DOWN
89	P00450	0.109	DOWN	0.152	DOWN	0.155	DOWN
90	A0A0A0MRZ8	0.408	DOWN	0.465	DOWN	0.155	DOWN
91	P0DOY3	0.128	DOWN	0.347	DOWN	0.155	DOWN
92	A0A0A0MS15	0.333	DOWN	0.462	DOWN	0.158	DOWN
93	P01717	0.221	DOWN	**	UP	0.158	DOWN
94	P21926	0.116	DOWN	0.021	DOWN	0.186	DOWN
95	A0A075B6P5	**	UP	0.341	DOWN	0.215	DOWN
96	P00488	0.173	DOWN	0.030	DOWN	0.221	DOWN
97	P14618	0.304	DOWN	0.033	DOWN	0.227	DOWN
98	P62937	0.341	DOWN	0.038	DOWN	0.249	DOWN
99	P11226	0.772	[OT]	0.499	DOWN	0.255	DOWN
100	P01619	0.477	DOWN	0.644	[OT]	0.255	DOWN
101	P80748	**	UP	**	UP	0.269	DOWN
102	P01591	0.516	[OT]	0.191	DOWN	0.299	DOWN
103	P01701	3.072	UP	3.584	UP	0.315	DOWN
104	A0A0C4DH68	**	UP	0.417	DOWN	0.325	DOWN
105	P20742	**	UP	0.052	DOWN	0.341	DOWN
106	P07195	1.639	[OT]	0.040	DOWN	0.362	DOWN
107	P06312	0.236	DOWN	0.538	[OT]	0.376	DOWN
108	P04406	0.195	DOWN	0.039	DOWN	0.388	DOWN
109	P01700	1.377	[OT]	1.736	[OT]	0.406	DOWN
110	P11021	1.149	[OT]	0.045	DOWN	0.418	DOWN
111	P35908	**	UP	**	UP	0.420	DOWN
112	P06331	0.233	DOWN	1.593	[OT]	0.426	DOWN

113	P13645	7.220	UP	0.514	[OT]	0.436	DOWN
114	P01703	**	UP	2.455	UP	0.445	DOWN
115	P02748	0.663	[OT]	0.431	DOWN	0.445	DOWN
116	P35858	0.253	DOWN	0.236	DOWN	0.460	DOWN
117	P06702	**	UP	**	UP	0.471	DOWN
118	P04075	0.416	DOWN	0.038	DOWN	0.475	DOWN
119	P02671	2.061	UP	0.152	DOWN	0.519	[OT]
120	O43866	1.142	[OT]	0.224	DOWN	0.541	[OT]
121	O14791	0.315	DOWN	0.347	DOWN	0.546	[OT]
122	P02750	2.478	UP	0.692	[OT]	0.600	[OT]
123	P01871	1.073	[OT]	0.257	DOWN	0.615	[OT]
124	P04264	3.929	UP	0.190	DOWN	0.659	[OT]
125	P01780	0.481	DOWN	0.635	[OT]	0.671	[OT]
126	P02675	2.087	UP	0.225	DOWN	0.693	[OT]
127	P02768	0.738	[OT]	12.696	UP	0.711	[OT]
128	P08571	0.543	[OT]	0.433	DOWN	0.725	[OT]
129	P02743	0.289	DOWN	0.521	[OT]	0.731	[OT]
130	A0A0B4J1X5	0.799	[OT]	0.483	DOWN	0.746	[OT]
131	P18428	0.714	[OT]	**	UP	0.756	[OT]
132	P01009	0.821	[OT]	0.644	[OT]	0.768	[OT]
133	P02679	1.967	[OT]	0.228	DOWN	0.775	[OT]
134	P02652	0.783	[OT]	0.991	[OT]	0.798	[OT]
135	P05090	0.385	DOWN	0.925	[OT]	0.803	[OT]
136	Q13201	0.790	[OT]	0.511	[OT]	0.806	[OT]
137	P02749	1.315	[OT]	1.314	[OT]	0.812	[OT]
138	P07358	1.079	[OT]	0.785	[OT]	0.834	[OT]
139	P07357	1.119	[OT]	0.849	[OT]	0.848	[OT]
140	P00740	0.735	[OT]	0.607	[OT]	0.853	[OT]
141	Q13790	2.230	UP	0.563	[OT]	0.854	[OT]
142	P04004	0.800	[OT]	0.969	[OT]	0.858	[OT]
143	Q14624	1.059	[OT]	0.735	[OT]	0.870	[OT]
144	P02654	1.437	[OT]	1.222	[OT]	0.876	[OT]
145	P19823	1.094	[OT]	0.607	[OT]	0.878	[OT]
146	P00338	0.247	DOWN	0.046	DOWN	0.878	[OT]
147	P14625	0.352	DOWN	0.131	DOWN	0.888	[OT]
148	A0A0B4J1V0	**	UP	4.299	UP	0.893	[OT]
149	Q06033	2.437	UP	0.979	[OT]	0.895	[OT]
150	P06727	0.903	[OT]	1.129	[OT]	0.896	[OT]
151	P00751	1.127	[OT]	0.902	[OT]	0.918	[OT]
152	P00742	0.535	[OT]	1.005	[OT]	0.928	[OT]
153	P01011	0.986	[OT]	0.975	[OT]	0.939	[OT]
154	O75636	0.530	[OT]	0.866	[OT]	0.943	[OT]
155	P19652	0.730	[OT]	0.785	[OT]	0.958	[OT]
156	P22352	0.433	DOWN	0.616	[OT]	0.967	[OT]
157	P02763	0.821	[OT]	1.148	[OT]	0.981	[OT]
158	P19827	0.997	[OT]	0.645	[OT]	0.983	[OT]
159	P02647	0.790	[OT]	1.262	[OT]	0.995	[OT]
160	P25311	0.820	[OT]	0.900	[OT]	0.997	[OT]
161	P01024	0.832	[OT]	0.912	[OT]	0.999	[OT]
162	P02656	0.970	[OT]	1.184	[OT]	1.001	[OT]
163	P27918	11.214	UP	0.953	[OT]	1.008	[OT]
164	P02746	0.723	[OT]	0.703	[OT]	1.010	[OT]
165	Q15848	1.535	[OT]	0.707	[OT]	1.013	[OT]
166	P35527	**	UP	0.912	[OT]	1.027	[OT]
167	P36955	0.928	[OT]	0.974	[OT]	1.028	[OT]
168	P02042	0.502	[OT]	5.093	UP	1.032	[OT]
169	P05160	1.779	[OT]	0.260	DOWN	1.037	[OT]
170	P02747	1.299	[OT]	0.603	[OT]	1.073	[OT]

171	P05543	1.357	[OT]	1.217	[OT]	1.078	[OT]
172	P00747	1.343	[OT]	0.704	[OT]	1.081	[OT]
173	Q12805	0.453	DOWN	0.357	DOWN	1.090	[OT]
174	P06396	0.747	[OT]	0.530	[OT]	1.106	[OT]
175	P09871	0.843	[OT]	0.754	[OT]	1.110	[OT]
176	P04275	2.300	UP	0.344	DOWN	1.112	[OT]
177	P06733	1.473	[OT]	0.107	DOWN	1.119	[OT]
178	P05156	1.288	[OT]	1.206	[OT]	1.172	[OT]
179	P02760	1.502	[OT]	0.854	[OT]	1.175	[OT]
180	O75882	1.569	[OT]	1.183	[OT]	1.192	[OT]
181	Q9UK55	1.461	[OT]	1.039	[OT]	1.193	[OT]
182	Q14520	2.746	UP	0.829	[OT]	1.202	[OT]
183	P02751	2.926	UP	0.342	DOWN	1.215	[OT]
184	P02649	1.078	[OT]	1.720	[OT]	1.234	[OT]
185	P00915	0.530	[OT]	0.956	[OT]	1.250	[OT]
186	P0C0L4	0.348	DOWN	1.663	[OT]	1.256	[OT]
187	Q08380	13.423	UP	0.936	[OT]	1.261	[OT]
188	P04217	2.074	UP	1.061	[OT]	1.269	[OT]
189	P07360	1.477	[OT]	1.014	[OT]	1.298	[OT]
190	Q9UGM5	**	UP	3.322	UP	1.301	[OT]
191	P00738	0.915	[OT]	0.511	[OT]	1.307	[OT]
192	P01031	0.778	[OT]	1.086	[OT]	1.310	[OT]
193	P02766	0.967	[OT]	0.991	[OT]	1.336	[OT]
194	P48740	1.727	[OT]	1.837	[OT]	1.342	[OT]
195	P01008	1.263	[OT]	1.260	[OT]	1.359	[OT]
196	P10909	1.514	[OT]	1.016	[OT]	1.380	[OT]
197	P08603	1.745	[OT]	1.216	[OT]	1.382	[OT]
198	P02790	1.725	[OT]	1.129	[OT]	1.383	[OT]
199	P43251	1.257	[OT]	1.080	[OT]	1.389	[OT]
200	P01023	1.773	[OT]	1.388	[OT]	1.414	[OT]
201	P49908	1.264	[OT]	1.452	[OT]	1.415	[OT]
202	P22792	1.187	[OT]	1.051	[OT]	1.422	[OT]
203	P02787	1.028	[OT]	1.194	[OT]	1.450	[OT]
204	P09211	**	UP	0.285	DOWN	1.460	[OT]
205	P61769	2.956	UP	0.471	DOWN	1.468	[OT]
206	P08697	1.739	[OT]	1.003	[OT]	1.477	[OT]
207	Q92954	0.511	[OT]	1.188	[OT]	1.512	UP
208	P27169	0.971	[OT]	1.427	[OT]	1.535	UP
209	P07359	1.253	[OT]	0.621	[OT]	1.586	UP
210	P20851	3.685	UP	1.031	[OT]	1.589	UP
211	P00734	2.476	UP	1.117	[OT]	1.591	UP
212	P01042	1.368	[OT]	1.347	[OT]	1.609	UP
213	P05155	1.944	[OT]	1.556	[OT]	1.614	UP
214	P02774	1.569	[OT]	1.510	[OT]	1.622	UP
215	Q9NP78	**	UP	0.567	[OT]	1.622	UP
216	P04114	1.196	[OT]	1.382	[OT]	1.625	UP
217	P51884	4.767	UP	1.995	[OT]	1.626	UP
218	P02775	1.405	[OT]	0.637	[OT]	1.627	UP
219	P08185	1.201	[OT]	1.042	[OT]	1.635	UP
220	P02765	1.789	[OT]	1.636	[OT]	1.643	UP
221	Q16610	3.366	UP	0.718	[OT]	1.685	UP
222	P29622	0.779	[OT]	1.087	[OT]	1.689	UP
223	P00736	1.736	[OT]	1.154	[OT]	1.694	UP
224	P43652	1.817	[OT]	1.438	[OT]	1.694	UP
225	P04003	1.599	[OT]	1.550	[OT]	1.701	UP
226	P02745	1.487	[OT]	1.130	[OT]	1.715	UP
227	P02655	4.787	UP	1.365	[OT]	1.721	UP
228	P22105	**	UP	**	UP	1.731	UP

229	P00739	1.513	[OT]	1.032	[OT]	1.736	UP
230	Q96IY4	1.584	[OT]	1.061	[OT]	1.741	UP
231	P05546	1.408	[OT]	1.180	[OT]	1.755	UP
232	P12259	**	UP	0.588	[OT]	1.757	UP
233	P05452	1.103	[OT]	1.422	[OT]	1.757	UP
234	O00391	**	UP	0.982	[OT]	1.776	UP
235	P07225	1.414	[OT]	1.035	[OT]	1.780	UP
236	P0C0L5	1.120	[OT]	1.271	[OT]	1.861	UP
237	P13671	2.440	UP	1.411	[OT]	1.910	UP
238	P00748	3.268	UP	1.309	[OT]	1.916	UP
239	P23142	4.292	UP	0.715	[OT]	1.921	UP
240	P02753	0.806	[OT]	1.292	[OT]	1.968	UP
241	P10643	4.020	UP	1.915	[OT]	2.014	UP
242	Q9NZP8	6.656	UP	1.946	[OT]	2.048	UP
243	Q04756	6.000	UP	3.180	UP	2.073	UP
244	P05154	1.418	[OT]	1.412	[OT]	2.117	UP
245	P03952	1.515	[OT]	1.104	[OT]	2.126	UP
246	P35542	1.803	[OT]	1.317	[OT]	2.131	UP
247	P00746	4.230	UP	1.651	[OT]	2.143	UP
248	O95445	1.778	[OT]	2.221	UP	2.149	UP
249	P01019	1.384	[OT]	2.183	UP	2.155	UP
250	P15169	4.694	UP	1.625	[OT]	2.229	UP
251	Q96PD5	4.361	UP	1.430	[OT]	2.238	UP
252	P55056	2.703	UP	0.960	[OT]	2.276	UP
253	Q6UXB8	3.233	UP	2.512	UP	2.376	UP
254	P04070	**	UP	1.379	[OT]	2.394	UP
255	P80108	1.225	[OT]	1.492	[OT]	2.414	UP
256	P04196	1.792	[OT]	1.766	[OT]	2.491	UP
257	P03951	1.293	[OT]	2.384	UP	2.530	UP
258	P06276	2.608	UP	1.236	[OT]	2.578	UP
259	P68871	2.327	UP	3.990	UP	2.619	UP
260	P54108	**	UP	0.816	[OT]	2.865	UP
261	P00918	1.859	[OT]	0.432	DOWN	2.947	UP
262	P02776	2.494	UP	0.676	[OT]	3.007	UP
263	P26927	7.696	UP	2.633	UP	3.016	UP
264	P05109	1.132	[OT]	13.757	UP	3.047	UP
265	P07996	5.342	UP	1.322	[OT]	3.065	UP
266	P00441	**	UP	0.321	DOWN	3.118	UP
267	Q03591	2.841	UP	1.477	[OT]	3.148	UP
268	P04180	3.983	UP	1.496	[OT]	3.421	UP
269	P04278	**	UP	2.532	UP	3.867	UP
270	P04040	2.290	UP	0.949	[OT]	4.107	UP
271	P13473	2.597	UP	5.707	UP	4.372	UP
272	Q6EMK4	**	UP	1.435	[OT]	4.840	UP
273	O00187	**	UP	6.633	UP	6.282	UP
274	P13598	**	UP	0.906	[OT]	7.326	UP
275	P22891	**	UP	1.782	[OT]	7.367	UP
276	P14151	1.523	[OT]	1.124	[OT]	7.434	UP
277	P32119	7.476	UP	2.885	UP	7.543	UP
278	P61626	**	UP	1.995	[OT]	7.765	UP
279	P69892	5.582	UP	15.346	UP	12.279	UP
280	Q13103	10.618	UP	1.141	[OT]	12.663	UP
281	P55072	-	-	*	DOWN	-	-
282	P07384	-	-	*	DOWN	-	-
283	P0CG39	-	-	*	DOWN	-	-
284	P14543	-	-	*	DOWN	-	-
285	P16109	-	-	*	DOWN	-	-
286	P50395	-	-	*	DOWN	-	-

287	Q14247	-	-	*	DOWN	-	-
288	Q15084	-	-	*	DOWN	-	-
289	Q9UBW5	-	-	*	DOWN	-	-
290	Q92496	**	UP	1.437	[OT]	**	UP
291	Q15833	**	UP	163.695	UP	**	UP

*Non detected in proband

**Non detected in the reference (father, mother or healthy children control)

[OT] = Outside the treshold

UP= Upregulated

DOWN =Downregulated

Supplementary Table S2. Result table of gene ontology analysis for downregulated proteins considering the cellular component as classificator.

GO ID	Term	P-value	Uncorrected P-value	Number annotated	Annotated genes
GO:0070062	extracellular exosome	7.12E-30	3.34E-32	45	Actb, ACTN1, ALDOA, ARHGDIB, ARPC1B, CALR, CAP1, CD9, CFL1, CP, FERMT3, FLNA, GAPDH, GP5, HSPA1B, IGFALS, IGHA1, IGHA2, IGHD, IGHG2, IGHG3, IGHG4, IGKC, IGLL5, ITGA2B, ITGB3, Msn, MYH9, MYL12A, PDIA3, PFN1, PKM, PNP, PPIA, PRDX6, RAP1B, RSU1, TAGLN2, TLN1, TPM3, TPM4, TUBA4A, VCL, WDR1, YWHAZ
GO:1903561	extracellular vesicle	1.10E-29	5.18E-32	45	Actb, ACTN1, ALDOA, ARHGDIB, ARPC1B, CALR, CAP1, CD9, CFL1, CP, FERMT3, FLNA, GAPDH, GP5, HSPA1B, IGFALS, IGHA1, IGHA2, IGHD, IGHG2, IGHG3, IGHG4, IGKC, IGLL5, ITGA2B, ITGB3, Msn, MYH9, MYL12A, PDIA3, PFN1, PKM, PNP, PPIA, PRDX6, RAP1B, RSU1, TAGLN2, TLN1, TPM3, TPM4, TUBA4A, VCL, WDR1, YWHAZ
GO:0043230	extracellular organelle	1.22E-29	5.74E-32	45	Actb, ACTN1, ALDOA, ARHGDIB, ARPC1B, CALR, CAP1, CD9, CFL1, CP, FERMT3, FLNA, GAPDH, GP5, HSPA1B, IGFALS, IGHA1, IGHA2, IGHD, IGHG2, IGHG3, IGHG4, IGKC, IGLL5, ITGA2B, ITGB3, Msn, MYH9, MYL12A, PDIA3, PFN1, PKM, PNP, PPIA, PRDX6, RAP1B, RSU1, TAGLN2, TLN1, TPM3, TPM4, TUBA4A, VCL, WDR1, YWHAZ
GO:0005615	extracellular space	3.03E-26	1.42E-28	49	Actb, ACTN1, ALDOA, ARHGDIB, ARPC1B, CALR, CAP1, CD9, CFL1, CP, F13A1, FERMT3, FLNA, GAPDH, GP5, HSPA1B, IGFALS, IGHA1, IGHA2, IGHD, IGHG2, IGHG3, IGHG4, IGHV3-49, IGKC, IGKV3D-11, IGKV3D-7, IGLL5, ITGA2B, ITGB3, Msn, MYH9, MYL12A, PDIA3, PFN1, PKM, PNP, PPIA, PRDX6, RAP1B, RSU1, TAGLN2, TLN1, TPM3, TPM4, TUBA4A, VCL, WDR1, YWHAZ
GO:0044421	extracellular region part	5.56E-25	2.61E-27	49	Actb, ACTN1, ALDOA, ARHGDIB, ARPC1B, CALR, CAP1, CD9, CFL1, CP, F13A1, FERMT3, FLNA, GAPDH, GP5, HSPA1B, IGFALS, IGHA1, IGHA2, IGHD, IGHG2, IGHG3, IGHG4, IGHV3-49, IGKC, IGKV3D-11, IGKV3D-7, IGLL5, ITGA2B, ITGB3, Msn, MYH9, MYL12A, PDIA3, PFN1, PKM, PNP, PPIA, PRDX6, RAP1B, RSU1, TAGLN2, TLN1, TPM3, TPM4, TUBA4A, VCL, WDR1, YWHAZ
GO:0031982	vesicle	1.71E-24	8.03E-27	50	Actb, ACTN1, ALDOA, ARHGDIB, ARPC1B, BTK, CALR, CAP1, CD9, CFL1, CORO1C, CP, F13A1, FERMT3, FLNA, GAPDH, GP5, HSPA1B, IGFALS, IGHA1, IGHA2, IGHD, IGHG2, IGHG3, IGHG4, IGKC, IGLL5, ITGA2B, ITGB3, Msn, MYH9, MYL12A, PDIA3, PFN1, PKM, PNP, PPIA, PRDX6, RAP1B, RSU1, TAGLN2, TLN1, TMSB4X, TPM3, TPM4, TUBA1C, TUBA4A, VCL, WDR1, YWHAZ
GO:0005576	extracellular region	2.97E-23	1.40E-25	51	Actb, ACTN1, ALDOA, ARHGDIB, ARPC1B, CALR, CAP1, CD9, CFL1, CP, F13A1, FERMT3, FLNA, GAPDH, GP5, HSPA1B, IGFALS, IGHA1, IGHA2, IGHD, IGHG2, IGHG3, IGHG4, IGHV3-49, IGKC, IGKV3D-11, IGKV3D-7, IGLL5, ITGA2B, ITGB3, Msn, MYH9, MYL12A, PDIA3, PFN1, PKM, PLEK, PNP, PPIA, PRDX6, RAP1B, RSU1, TAGLN2, TLN1, TMSB4X, TPM3, TPM4, TUBA4A, VCL, WDR1, YWHAZ
GO:0005925	focal adhesion	1.60E-21	7.52E-24	23	Actb, ACTN1, ARPC1B, CALR, CAP1, CD9, CFL1, CORO1C, FLNA, HSPA1B, ILK, ITGA2B, ITGB3, Msn, MYH9, PDIA3, PFN1, PPIA, RSU1, TLN1, TPM4, VCL, YWHAZ
GO:0005924	cell-substrate adherens junction	1.90E-21	8.90E-24	23	Actb, ACTN1, ARPC1B, CALR, CAP1, CD9, CFL1, CORO1C, FLNA, HSPA1B, ILK, ITGA2B, ITGB3, Msn, MYH9, PDIA3, PFN1, PPIA, RSU1, TLN1, TPM4, VCL, YWHAZ
GO:0030055	cell-substrate junction	2.37E-21	1.11E-23	23	Actb, ACTN1, ARPC1B, CALR, CAP1, CD9, CFL1, CORO1C, FLNA, HSPA1B, ILK, ITGA2B, ITGB3, Msn, MYH9, PDIA3, PFN1, PPIA, RSU1, TLN1, TPM4, VCL, YWHAZ
GO:0005912	adherens junction	1.25E-18	5.85E-21	23	Actb, ACTN1, ARPC1B, CALR, CAP1, CD9, CFL1, CORO1C, FLNA, HSPA1B, ILK, ITGA2B, ITGB3, Msn, MYH9, PDIA3, PFN1, PPIA, RSU1, TLN1, TPM4, VCL, YWHAZ
GO:0070161	anchoring junction	2.38E-18	1.12E-20	23	Actb, ACTN1, ARPC1B, CALR, CAP1, CD9, CFL1, CORO1C, FLNA, HSPA1B, ILK, ITGA2B, ITGB3, Msn, MYH9, PDIA3, PFN1, PPIA, RSU1, TLN1, TPM4, VCL, YWHAZ
GO:0072562	blood microparticle	2.54E-17	1.19E-19	15	Actb, CP, F13A1, HSPA1B, IGHA1, IGHA2, IGHD, IGHG2, IGHG3, IGHG4, IGKC, ITGA2B, Msn, PFN1, YWHAZ
GO:0030054	cell junction	4.55E-13	2.13E-15	26	Actb, ACTN1, ARPC1B, CALR, CAP1, CD9, CFL1, CORO1C, FERMT3, FLNA, HSPA1B, ILK, ITGA2B, ITGB3, Msn, MYH9, PDIA3, PFN1, PPIA, RAP1B, RSU1, TLN1, TPM4, VCL, WDR1, YWHAZ
GO:0015629	actin cytoskeleton	2.37E-11	1.11E-13	17	Actb, ACTN1, ALDOA, ARPC1B, CAP1, CFL1, CORO1C, FERMT3, FLNA, ILK, MYH9, MYL12A, TPM1, TPM3, TPM4, VCL, WDR1
GO:0019814	immunoglobulin complex	1.60E-09	7.53E-12	11	IGHA1, IGHA2, IGHD, IGHG2, IGHG3, IGHG4, IGHV3-49, IGKC, IGKV3D-11, IGKV3D-7, IGLL5
GO:0042571	immunoglobulin complex, circulating	1.92E-09	9.03E-12	9	IGHA1, IGHA2, IGHD, IGHG2, IGHG3, IGHG4, IGHV3-49, IGKC, IGLL5
GO:0005856	cytoskeleton	9.83E-09	4.62E-11	27	Actb, ACTN1, ALDOA, ARHGDIB, ARPC1B, CAP1, CFL1, CORO1C, FERMT3, FLNA, GAPDH, HSPA1B, ILK, Msn, MYH9, MYL12A, PFN1, PNP, TLN1, TMSB4X, TPM1, TPM3, TPM4, TUBA1C, TUBA4A, VCL, WDR1
GO:0030141	secretory granule	5.01E-08	2.35E-10	17	ACTN1, ALDOA, CALR, CAP1, CD9, F13A1, FERMT3, HSPA1B, ITGA2B, ITGB3, PKM, PNP, PPIA, PRDX6, RAP1B, TMSB4X, VCL
GO:0060205	cytoplasmic vesicle lumen	5.86E-08	2.75E-10	12	ACTN1, ALDOA, CALR, CAP1, F13A1, FERMT3, PKM, PNP, PPIA, PRDX6, TMSB4X, VCL
GO:0031091	platelet alpha granule	5.95E-08	2.79E-10	8	ACTN1, ALDOA, CD9, F13A1, FERMT3, ITGA2B, ITGB3, TMSB4X
GO:0031983	vesicle lumen	6.06E-08	2.85E-10	12	ACTN1, ALDOA, CALR, CAP1, F13A1, FERMT3, PKM, PNP, PPIA, PRDX6, TMSB4X, VCL
GO:0034774	secretory granule lumen	5.27E-07	2.47E-09	11	ACTN1, ALDOA, CAP1, F13A1, FERMT3, PKM, PNP, PPIA, PRDX6, TMSB4X, VCL
GO:0099503	secretory vesicle	7.86E-07	3.69E-09	17	ACTN1, ALDOA, CALR, CAP1, CD9, F13A1, FERMT3, HSPA1B, ITGA2B, ITGB3, PKM, PNP, PPIA, PRDX6, RAP1B, TMSB4X, VCL
GO:0042641	actomyosin	8.60E-07	4.04E-09	7	ACTN1, ILK, MYH9, TPM1, TPM3, TPM4, WDR1
GO:0009897	external side of plasma membrane	8.85E-07	4.16E-09	12	CALR, CD9, IGHA1, IGHA2, IGHD, IGHG2, IGHG3, IGHG4, IGHV3-49, IGKC, IGLL5, ITGA2B
GO:0009986	cell surface	2.15E-06	1.01E-08	16	CALR, CD9, IGHA1, IGHA2, IGHD, IGHG2, IGHG3, IGHG4, IGHV3-49, IGKC, IGLL5, ITGA2B, ITGB3, Msn, PDIA3, TLN1
GO:0044449	contractile fiber part	3.97E-06	1.86E-08	9	ACTN1, ALDOA, CORO1C, FLNA, ILK, TPM1, TPM3, TPM4, VCL
GO:0030016	myofibril	4.46E-06	2.09E-08	9	ACTN1, ALDOA, CORO1C, FLNA, ILK, TPM1, TPM3, TPM4, VCL
GO:0043292	contractile fiber	6.75E-06	3.17E-08	9	ACTN1, ALDOA, CORO1C, FLNA, ILK, TPM1, TPM3, TPM4, VCL
GO:0044433	cytoplasmic vesicle part	9.85E-06	4.62E-08	19	ACTN1, ALDOA, CALR, CAP1, CD9, CORO1C, F13A1, FERMT3, HSPA1B, ITGA2B, ITGB3, PDIA3, PKM, PNP, PPIA, PRDX6, RAP1B, TMSB4X, VCL
GO:0001726	ruffle	1.12E-05	5.24E-08	8	ACTN1, CFL1, CORO1C, ITGB3, MYH9, PLEK, TLN1, TPM1
GO:0001725	stress fiber	1.13E-05	5.30E-08	6	ACTN1, ILK, MYH9, TPM1, TPM3, TPM4
GO:0097517	contractile actin filament bundle	1.13E-05	5.30E-08	6	ACTN1, ILK, MYH9, TPM1, TPM3, TPM4
GO:0005884	actin filament	1.55E-05	7.30E-08	7	Actb, ACTN1, FLNA, TPM1, TPM3, TPM4, WDR1
GO:0032432	actin filament bundle	2.04E-05	9.59E-08	6	ACTN1, ILK, MYH9, TPM1, TPM3, TPM4
GO:0005862	muscle thin filament tropomyosin	2.18E-05	1.02E-07	3	TPM1, TPM3, TPM4
GO:0030017	sarcomere	3.49E-05	1.64E-07	8	ACTN1, ALDOA, CORO1C, FLNA, ILK, TPM1, TPM3, TPM4
GO:0071944	cell periphery	3.72E-05	1.74E-07	37	Actb, ACTN1, BTK, CALR, CAP1, CD9, CFL1, CORO1C, CP, FLNA, GAPDH, GP5, HSPA1B, IGHA1, IGHA2, IGHD, IGHG2, IGHG3, IGHG4, IGHV3-49, IGKC, IGKV3D-11, IGKV3D-7, IGLL5, ILK, ITGA2B, ITGB3, Msn, MYH9, PFN1, PLEK, RAP1B, SDPR, TLN1, TPM1, VCL, WDR1
GO:0098552	side of membrane	5.77E-05	2.71E-07	12	CALR, CD9, IGHA1, IGHA2, IGHD, IGHG2, IGHG3, IGHG4, IGHV3-49, IGKC, IGLL5, ITGA2B
GO:0005829	cytosol	6.95E-05	3.26E-07	34	Actb, ACTN1, ALDOA, ARHGDIB, ARPC1B, BTK, CALR, CFL1, FLNA, GAPDH, HSPA1B, ILK, Msn, MYH9, MYL12A, PFN1, PKM, PLEK, PNP, PPIA, PRDX6, RAP1B, RSU1, SDPR, TAGLN2, TLN1, TMSB4X, TPM1, TPM3, TPM4, TUBA4A, VCL, WDR1, YWHAZ
GO:0032587	ruffle membrane	7.91E-05	3.71E-07	6	CFL1, CORO1C, ITGB3, PLEK, TLN1, TPM1
GO:0005886	plasma membrane	8.09E-05	3.80E-07	36	Actb, ACTN1, BTK, CALR, CAP1, CD9, CFL1, CORO1C, CP, FLNA, GAPDH, GP5, HSPA1B, IGHA1, IGHA2, IGHD, IGHG2, IGHG3, IGHG4, IGHV3-49, IGKC, IGKV3D-11, IGKV3D-7, IGLL5, ILK, ITGA2B, ITGB3, Msn, MYH9, PLEK, RAP1B, SDPR, TLN1, TPM1, VCL, WDR1
GO:0031252	cell leading edge	8.68E-05	4.07E-07	10	Actb, ACTN1, CFL1, CORO1C, ILK, ITGB3, MYH9, PLEK, TLN1, TPM1
GO:0031410	cytoplasmic vesicle	0.000128874	6.05E-07	22	ACTN1, ALDOA, ARHGDIB, BTK, CALR, CAP1, CD9, CORO1C, F13A1, FERMT3, HSPA1B, ITGA2B, ITGB3, PDIA3, PKM, PNP, PPIA, PRDX6, RAP1B, TMSB4X, VCL, YWHAZ
GO:0097708	intracellular vesicle	0.000131714	6.18E-07	22	ACTN1, ALDOA, ARHGDIB, BTK, CALR, CAP1, CD9, CORO1C, F13A1, FERMT3, HSPA1B, ITGA2B, ITGB3, PDIA3, PKM, PNP, PPIA, PRDX6, RAP1B, TMSB4X, VCL, YWHAZ
GO:0030863	cortical cytoskeleton	0.000288044	1.35E-06	6	Actb, CAP1, CFL1, FLNA, MYH9, WDR1
GO:0031093	platelet alpha granule lumen	0.000363446	1.71E-06	5	ACTN1, ALDOA, F13A1, FERMT3, TMSB4X
GO:0044430	cytoskeletal part	0.000398276	1.87E-06	18	Actb, ACTN1, ARPC1B, CAP1, CFL1, FERMT3, FLNA, HSPA1B, ILK, MYH9, MYL12A, TPM1, TPM3, TPM4, TUBA1C, TUBA4A, VCL, WDR1
GO:1904813	ficolin-1-rich granule lumen	0.000404662	1.90E-06	6	ALDOA, HSPA1B, PKM, PNP, PPIA, VCL
GO:0005911	cell-cell junction	0.000554672	2.60E-06	9	Actb, ACTN1, CFL1, FLNA, ILK, ITGB3, RAP1B, VCL, WDR1

Number of downregulated proteins equal to 59

The result table shows 59 downregulated proteins, 4 genes unannotated: No-name, calm1a, IGKV3D-11, and IGKV3D-7 ; 2 unkown: No-name and calm1a.

p-value ≤ 0.001

Supplementary Table S3 -Result table of gene ontology analysis for downregulated proteins considering the biological process as classificator.

GO ID	Term	P-value	Uncorrected P-value	Number annotated	Annotated genes
GO:0016192	vesicle-mediated transport	1.05E-19	9.40E-23	38	Actb, ACTN1, ALDOA, ARPC1B, BTK, CALR, CAP1, CD9, CORO1C, F13A1, FERMT3, FLNA, HSPA1B, IGHA1, IGHA2, IGHD, IGHG2, IGHG3, IGHG4, IGHV3-49, IGKC, IGLL5, ITGA2B, ITGB3, Msn, MYH9, PKM, PLEK, PNP, PPIA, PRDX6, RAP1B, TAGLN2, TLN1, TMSB4X, TUBA4A, VCL, WDR1
GO:0001775	cell activation	2.34E-19	2.08E-22	33	Actb, ALDOA, BTK, CAP1, CD9, FERMT3, FLNA, GP5, HSPA1B, IGHA1, IGHA2, IGHD, IGHG2, IGHG3, IGHG4, IGHV3-49, IGKC, IGLL5, ILK, ITGA2B, ITGB3, Msn, MYH9, MYL12A, PKM, PLEK, PNP, PPIA, PRDX6, RAP1B, TLN1, VCL, YWHAZ
GO:0002576	platelet degranulation	2.38E-17	2.12E-20	15	ACTN1, ALDOA, CD9, F13A1, FERMT3, FLNA, ITGA2B, ITGB3, PLEK, TAGLN2, TLN1, TMSB4X, TUBA4A, VCL, WDR1
GO:0070527	platelet aggregation	1.80E-16	1.61E-19	12	Actb, CD9, FERMT3, FLNA, ILK, ITGA2B, ITGB3, MYH9, MYL12A, PLEK, TLN1, VCL
GO:0045055	regulated exocytosis	1.57E-15	1.40E-18	24	ACTN1, ALDOA, BTK, CAP1, CD9, F13A1, FERMT3, FLNA, HSPA1B, ITGA2B, ITGB3, MYH9, PKM, PLEK, PNP, PPIA, PRDX6, RAP1B, TAGLN2, TLN1, TMSB4X, TUBA4A, VCL, WDR1
GO:0034109	homotypic cell-cell adhesion	1.48E-14	1.32E-17	12	Actb, CD9, FERMT3, FLNA, ILK, ITGA2B, ITGB3, MYH9, MYL12A, PLEK, TLN1, VCL
GO:0030168	platelet activation	1.78E-14	1.58E-17	14	Actb, CD9, FERMT3, FLNA, GP5, ILK, ITGA2B, ITGB3, MYH9, MYL12A, PLEK, TLN1, VCL, YWHAZ
GO:0006887	exocytosis	3.03E-14	2.70E-17	24	ACTN1, ALDOA, BTK, CAP1, CD9, F13A1, FERMT3, FLNA, HSPA1B, ITGA2B, ITGB3, MYH9, PKM, PLEK, PNP, PPIA, PRDX6, RAP1B, TAGLN2, TLN1, TMSB4X, TUBA4A, VCL, WDR1
GO:0030036	actin cytoskeleton organization	4.94E-12	4.40E-15	20	Actb, ACTN1, ALDOA, ARHGDIB, ARPC1B, CALR, CAP1, CFL1, CORO1C, FLNA, ILK, MYH9, PFN1, PLEK, TLN1, TMSB4X, TPM1, TPM3, TPM4, WDR1
GO:0007596	blood coagulation	2.85E-11	2.54E-14	15	Actb, CD9, F13A1, FERMT3, FLNA, GP5, ILK, ITGA2B, ITGB3, MYH9, MYL12A, PLEK, TLN1, VCL, YWHAZ
GO:0050817	coagulation	3.41E-11	3.04E-14	15	Actb, CD9, F13A1, FERMT3, FLNA, GP5, ILK, ITGA2B, ITGB3, MYH9, MYL12A, PLEK, TLN1, VCL, YWHAZ
GO:0007010	cytoskeleton organization	3.48E-11	3.10E-14	25	Actb, ACTN1, ALDOA, ARHGDIB, ARPC1B, CALR, CAP1, CFL1, CORO1C, FLNA, GAPDH, HSPA1B, ILK, Msn, MYH9, PFN1, PLEK, TLN1, TMSB4X, TPM1, TPM3, TPM4, TUBA1C, TUBA4A, WDR1
GO:0007599	hemostasis	3.57E-11	3.18E-14	15	Actb, CD9, F13A1, FERMT3, FLNA, GP5, ILK, ITGA2B, ITGB3, MYH9, MYL12A, PLEK, TLN1, VCL, YWHAZ
GO:0032940	secretion by cell	3.83E-11	3.42E-14	26	ACTN1, ALDOA, BTK, CAP1, CD9, F13A1, FERMT3, FLNA, GAPDH, HSPA1B, IGHD, ITGA2B, ITGB3, MYH9, PKM, PLEK, PNP, PPIA, PRDX6, RAP1B, TAGLN2, TLN1, TMSB4X, TUBA4A, VCL, WDR1
GO:0030029	actin filament-based process	5.64E-11	5.03E-14	20	Actb, ACTN1, ALDOA, ARHGDIB, ARPC1B, CALR, CAP1, CFL1, CORO1C, FLNA, ILK, MYH9, PFN1, PLEK, TLN1, TMSB4X, TPM1, TPM3, TPM4, WDR1
GO:0002376	immune system process	9.44E-11	8.41E-14	35	Actb, ACTN1, ALDOA, ARPC1B, BTK, CALR, CAP1, CD9, FLNA, GAPDH, HSPA1B, IGHA1, IGHA2, IGHD, IGHG2, IGHG3, IGHG4, IGHV3-49, IGKC, IGKV3D-11, IGKV3D-7, IGLL5, ITGA2B, ITGB3, Msn, MYH9, PDIA3, PKM, PLEK, PNP, PPIA, PRDX6, RAP1B, VCL, WDR1
GO:0008037	cell recognition	2.01E-10	1.79E-13	13	ALDOA, CD9, IGHA1, IGHA2, IGHD, IGHG2, IGHG3, IGHG4, IGHV3-49, IGKC, IGLL5, Msn, YWHAZ
GO:0046903	secretion	2.96E-10	2.64E-13	26	ACTN1, ALDOA, BTK, CAP1, CD9, F13A1, FERMT3, FLNA, GAPDH, HSPA1B, IGHD, ITGA2B, ITGB3, MYH9, PKM, PLEK, PNP, PPIA, PRDX6, RAP1B, TAGLN2, TLN1, TMSB4X, TUBA4A, VCL, WDR1
GO:0042060	wound healing	3.28E-10	2.92E-13	17	Actb, CD9, F13A1, FERMT3, FLNA, GP5, ILK, ITGA2B, ITGB3, MYH9, MYL12A, PKM, PLEK, TLN1, TPM1, VCL, YWHAZ
GO:0006909	phagocytosis	3.96E-10	3.53E-13	15	Actb, ARPC1B, CALR, CORO1C, IGHA1, IGHA2, IGHD, IGHG2, IGHG3, IGHG4, IGHV3-49, IGKC, IGLL5, ITGB3, MYH9
GO:0002443	leukocyte mediated immunity	8.66E-10	7.72E-13	20	ALDOA, BTK, CAP1, HSPA1B, IGHA1, IGHA2, IGHD, IGHG2, IGHG3, IGHG4, IGHV3-49, IGKC, IGLL5, PKM, PNP, PPIA, PRDX6, RAP1B, VCL, WDR1
GO:0002252	immune effector process	9.64E-10	8.59E-13	23	Actb, ALDOA, ARPC1B, BTK, CAP1, FLNA, HSPA1B, IGHA1, IGHA2, IGHD, IGHG2, IGHG3, IGHG4, IGHV3-49, IGKC, IGLL5, PKM, PNP, PPIA, PRDX6, RAP1B, VCL, WDR1
GO:0051179	localization	1.11E-09	9.90E-13	47	Actb, ACTN1, ALDOA, ARHGDIB, ARPC1B, BTK, CALR, CAP1, CD9, CFL1, CORO1C, CP, F13A1, FERMT3, FLNA, GAPDH, HSPA1B, IGHA1, IGHA2, IGHD, IGHG2, IGHG3, IGHG4, IGHV3-49, IGKC, IGLL5, ILK, ITGA2B, ITGB3, Msn, MYH9, PFN1, PKM, PLEK, PNP, PPIA, PRDX6, RAP1B, TAGLN2, TLN1, TMSB4X, TPM1, TUBA1C, TUBA4A, VCL, WDR1, YWHAZ
GO:0006810	transport	1.26E-09	1.13E-12	42	Actb, ACTN1, ALDOA, ARPC1B, BTK, CALR, CAP1, CD9, CORO1C, CP, F13A1, FERMT3, FLNA, GAPDH, HSPA1B, IGHA1, IGHA2, IGHD, IGHG2, IGHG3, IGHG4, IGHV3-49, IGKC, IGLL5, ITGA2B, ITGB3, Msn, MYH9, PKM, PLEK, PNP, PPIA, PRDX6, RAP1B, TAGLN2, TLN1, TMSB4X, TUBA1C, TUBA4A, VCL, WDR1, YWHAZ
GO:0051234	establishment of localization	2.96E-09	2.64E-12	42	Actb, ACTN1, ALDOA, ARPC1B, BTK, CALR, CAP1, CD9, CORO1C, CP, F13A1, FERMT3, FLNA, GAPDH, HSPA1B, IGHA1, IGHA2, IGHD, IGHG2, IGHG3, IGHG4, IGHV3-49, IGKC, IGLL5, ITGA2B, ITGB3, Msn, MYH9, PKM, PLEK, PNP, PPIA, PRDX6, RAP1B, TAGLN2, TLN1, TMSB4X, TUBA1C, TUBA4A, VCL, WDR1, YWHAZ
GO:0009611	response to wounding	6.01E-09	5.35E-12	17	Actb, CD9, F13A1, FERMT3, FLNA, GP5, ILK, ITGA2B, ITGB3, MYH9, MYL12A, PKM, PLEK, TLN1, TPM1, VCL, YWHAZ
GO:0050878	regulation of body fluid levels	1.23E-08	1.10E-11	15	Actb, CD9, F13A1, FERMT3, FLNA, GP5, ILK, ITGA2B, ITGB3, MYH9, MYL12A, PLEK, TLN1, VCL, YWHAZ
GO:0006911	phagocytosis, engulfment	1.35E-08	1.20E-11	10	IGHA1, IGHA2, IGHD, IGHG2, IGHG3, IGHG4, IGHV3-49, IGKC, IGLL5, MYH9
GO:0050853	B cell receptor signaling pathway	2.19E-08	1.95E-11	10	BTK, IGHA1, IGHA2, IGHD, IGHG2, IGHG3, IGHG4, IGHV3-49, IGKC, IGLL5
GO:0007015	actin filament organization	2.24E-08	2.00E-11	14	ACTN1, ALDOA, ARPC1B, CAP1, CFL1, CORO1C, FLNA, PFN1, PLEK, TMSB4X, TPM1, TPM3, TPM4, WDR1
GO:0099024	plasma membrane invagination	2.50E-08	2.23E-11	10	IGHA1, IGHA2, IGHD, IGHG2, IGHG3, IGHG4, IGHV3-49, IGKC, IGLL5, MYH9
GO:0006910	phagocytosis, recognition	3.04E-08	2.71E-11	9	IGHA1, IGHA2, IGHD, IGHG2, IGHG3, IGHG4, IGHV3-49, IGKC, IGLL5
GO:0010324	membrane invagination	4.20E-08	3.74E-11	10	IGHA1, IGHA2, IGHD, IGHG2, IGHG3, IGHG4, IGHV3-49, IGKC, IGLL5, MYH9
GO:0007155	cell adhesion	4.56E-08	4.06E-11	22	Actb, ACTN1, ARHGDIB, CALR, CD9, CORO1C, FERMT3, FLNA, GP5, IGFALS, ILK, ITGA2B, ITGB3, Msn, MYH9, MYL12A, PLEK, PNP, RSU1, TLN1, TPM1, VCL
GO:0072376	protein activation cascade	4.72E-08	4.20E-11	11	F13A1, GP5, IGHA1, IGHA2, IGHD, IGHG2, IGHG3, IGHG4, IGHV3-49, IGKC, IGLL5
GO:0022610	biological adhesion	5.02E-08	4.47E-11	22	Actb, ACTN1, ARHGDIB, CALR, CD9, CORO1C, FERMT3, FLNA, GP5, IGFALS, ILK, ITGA2B, ITGB3, Msn, MYH9, MYL12A, PLEK, PNP, RSU1, TLN1, TPM1, VCL
GO:0045321	leukocyte activation	7.59E-08	6.76E-11	21	ALDOA, BTK, CAP1, HSPA1B, IGHA1, IGHA2, IGHD, IGHG2, IGHG3, IGHG4, IGHV3-49, IGKC, IGLL5, Msn, MYH9, PKM, PNP, PPIA, PRDX6, RAP1B, VCL
GO:0050871	positive regulation of B cell activation	7.72E-08	6.88E-11	10	BTK, IGHA1, IGHA2, IGHD, IGHG2, IGHG3, IGHG4, IGHV3-49, IGKC, IGLL5
GO:0006928	movement of cell or subcellular component	9.55E-08	8.51E-11	26	Actb, ACTN1, ARHGDIB, CALR, CAP1, CD9, CFL1, CORO1C, FERMT3, FLNA, IGHA1, IGHA2, IGKC, ILK, ITGA2B, ITGB3, Msn, MYH9, PFN1, PPIA, TMSB4X, TPM1, TPM3, TPM4, VCL, WDR1
GO:0065008	regulation of biological quality	1.07E-07	9.51E-11	35	Actb, ALDOA, ARPC1B, BTK, CALR, CD9, CFL1, CP, F13A1, FERMT3, FLNA, GAPDH, GP5, HSPA1B, IGHA1, IGHA2, IGHG3, IGKC, ILK, ITGA2B, ITGB3, Msn, MYH9, MYL12A, PDIA3, PFN1, PLEK, PRDX6, RAP1B, TLN1, TMSB4X, TPM1, VCL, WDR1, YWHAZ

GO:0097435	supramolecular fiber organization	1.35E-07	1.21E-10	16	ACTN1, ALDOA, ARPC1B, CAP1, CFL1, CORO1C, FLNA, HSPA1B, ILK, PFN1, PLEK, TMSB4X, TPM1, TPM3, TPM4, WDR1
GO:0051674	localization of cell	1.48E-07	1.32E-10	23	Actb, ARHGDIB, CALR, CAP1, CD9, CFL1, CORO1C, FERMT3, FLNA, IGHA1, IGHA2, IGKC, ILK, ITGA2B, ITGB3, Msn, MYH9, PFN1, PPIA, TMSB4X, TPM1, VCL, WDR1
GO:0048870	cell motility	1.48E-07	1.32E-10	23	Actb, ARHGDIB, CALR, CAP1, CD9, CFL1, CORO1C, FERMT3, FLNA, IGHA1, IGHA2, IGKC, ILK, ITGA2B, ITGB3, Msn, MYH9, PFN1, PPIA, TMSB4X, TPM1, VCL, WDR1
GO:0016477	cell migration	1.48E-07	1.32E-10	22	ARHGDIB, CALR, CAP1, CD9, CFL1, CORO1C, FERMT3, FLNA, IGHA1, IGHA2, IGKC, ILK, ITGA2B, ITGB3, Msn, MYH9, PFN1, PPIA, TMSB4X, TPM1, VCL, WDR1
GO:0006897	endocytosis	2.04E-07	1.82E-10	17	Actb, ARPC1B, CALR, CAP1, CD9, CORO1C, IGHA1, IGHA2, IGHD, IGHG2, IGHG3, IGHG4, IGHV3-49, IGKC, IGLL5, ITGB3, MYH9
GO:0016043	cellular component organization	2.92E-07	2.60E-10	43	Actb, ACTN1, ALDOA, ARHGDIB, ARPC1B, CALR, CAP1, CD9, CFL1, CORO1C, FERMT3, FLNA, GAPDH, HSPA1B, IGHA1, IGHA2, IGHD, IGHG2, IGHG3, IGHG4, IGHV3-49, IGKC, IGLL5, ILK, ITGA2B, ITGB3, Msn, MYH9, PFN1, PLEK, PPIA, RAP1B, SDPR, TLN1, TMSB4X, TPM1, TPM3, TPM4, TUBA1C, TUBA4A, VCL, WDR1, YWHAZ
GO:0007166	cell surface receptor signaling pathway	4.82E-07	4.30E-10	30	Actb, ARPC1B, BTK, CFL1, F13A1, FERMT3, FLNA, HSPA1B, IGHA1, IGHA2, IGHD, IGHG2, IGHG3, IGHG4, IGHV3-49, IGKC, IGLL5, ILK, ITGA2B, ITGB3, Msn, MYH9, PDIA3, PFN1, PLEK, PPIA, RAP1B, TLN1, TMSB4X, YWHAZ
GO:0050864	regulation of B cell activation	7.23E-07	6.45E-10	10	BTK, IGHA1, IGHA2, IGHD, IGHG2, IGHG3, IGHG4, IGHV3-49, IGKC, IGLL5
GO:0071840	cellular component organization or biogenesis	8.02E-07	7.15E-10	43	Actb, ACTN1, ALDOA, ARHGDIB, ARPC1B, CALR, CAP1, CD9, CFL1, CORO1C, FERMT3, FLNA, GAPDH, HSPA1B, IGHA1, IGHA2, IGHD, IGHG2, IGHG3, IGHG4, IGHV3-49, IGKC, IGLL5, ILK, ITGA2B, ITGB3, Msn, MYH9, PFN1, PLEK, PPIA, RAP1B, SDPR, TLN1, TMSB4X, TPM1, TPM3, TPM4, TUBA1C, TUBA4A, VCL, WDR1, YWHAZ
GO:0061024	membrane organization	9.43E-07	8.41E-10	17	Actb, CALR, CD9, CORO1C, IGHA1, IGHA2, IGHD, IGHG2, IGHG3, IGHG4, IGHV3-49, IGKC, IGLL5, MYH9, PPIA, SDPR, YWHAZ
GO:0007229	integrin-mediated signaling pathway	1.07E-06	9.57E-10	8	FERMT3, FLNA, ILK, ITGA2B, ITGB3, MYH9, PLEK, TLN1
GO:0006958	complement activation, classical pathway	1.30E-06	1.16E-09	9	IGHA1, IGHA2, IGHD, IGHG2, IGHG3, IGHG4, IGHV3-49, IGKC, IGLL5
GO:0098657	import into cell	1.69E-06	1.51E-09	17	Actb, ARPC1B, CALR, CAP1, CD9, CORO1C, IGHA1, IGHA2, IGHD, IGHG2, IGHG3, IGHG4, IGHV3-49, IGKC, IGLL5, ITGB3, MYH9
GO:0040011	locomotion	1.89E-06	1.69E-09	23	Actb, ARHGDIB, CALR, CAP1, CD9, CFL1, CORO1C, FERMT3, FLNA, IGHA1, IGHA2, IGKC, ILK, ITGA2B, ITGB3, Msn, MYH9, PFN1, PPIA, TMSB4X, TPM1, VCL, WDR1
GO:0002455	humoral immune response mediated by circulating immunoglobulin	2.59E-06	2.31E-09	9	IGHA1, IGHA2, IGHD, IGHG2, IGHG3, IGHG4, IGHV3-49, IGKC, IGLL5
GO:0016064	immunoglobulin mediated immune response	3.07E-06	2.74E-09	10	BTK, IGHA1, IGHA2, IGHD, IGHG2, IGHG3, IGHG4, IGHV3-49, IGKC, IGLL5
GO:0034329	cell junction assembly	3.20E-06	2.85E-09	10	Actb, ACTN1, CD9, CORO1C, FLNA, ILK, RAP1B, TLN1, VCL, WDR1
GO:0019724	B cell mediated immunity	3.46E-06	3.08E-09	10	BTK, IGHA1, IGHA2, IGHD, IGHG2, IGHG3, IGHG4, IGHV3-49, IGKC, IGLL5
GO:0050867	positive regulation of cell activation	3.58E-06	3.19E-09	12	BTK, IGHA1, IGHA2, IGHD, IGHG2, IGHG3, IGHG4, IGHV3-49, IGKC, IGLL5, PLEK, PNP
GO:0006950	response to stress	3.85E-06	3.43E-09	33	Actb, BTK, CALR, CD9, F13A1, FERMT3, FLNA, GAPDH, GP5, HSPA1B, IGHA1, IGHA2, IGHD, IGHG2, IGHG3, IGHG4, IGHV3-49, IGKC, IGLL5, ILK, ITGA2B, ITGB3, MYH9, MYL12A, PDIA3, PKM, PLEK, PRDX6, TLN1, TMSB4X, TPM1, VCL, YWHAZ
GO:0006956	complement activation	7.04E-06	6.27E-09	9	IGHA1, IGHA2, IGHD, IGHG2, IGHG3, IGHG4, IGHV3-49, IGKC, IGLL5
GO:0051251	positive regulation of lymphocyte activation	1.00E-05	8.91E-09	11	BTK, IGHA1, IGHA2, IGHD, IGHG2, IGHG3, IGHG4, IGHV3-49, IGKC, IGLL5, PNP
GO:0006955	immune response	1.74E-05	1.55E-08	24	Actb, ALDOA, ARPC1B, BTK, CAP1, GAPDH, HSPA1B, IGHA1, IGHA2, IGHD, IGHG2, IGHG3, IGHG4, IGHV3-49, IGKC, IGKV3D-11, IGKV3D-7, IGLL5, PKM, PNP, PPIA, PRDX6, RAP1B, VCL
GO:0034330	cell junction organization	1.93E-05	1.72E-08	10	Actb, ACTN1, CD9, CORO1C, FLNA, ILK, RAP1B, TLN1, VCL, WDR1
GO:0002429	immune response-activating cell surface receptor signaling pathway	2.31E-05	2.05E-08	12	Actb, ARPC1B, BTK, IGHA1, IGHA2, IGHD, IGHG2, IGHG3, IGHG4, IGHV3-49, IGKC, IGLL5
GO:0032970	regulation of actin filament-based process	2.72E-05	2.42E-08	11	ARHGDIB, ARPC1B, CFL1, FLNA, ILK, MYH9, PFN1, PLEK, TMSB4X, TPM1, WDR1
GO:0002696	positive regulation of leukocyte activation	3.09E-05	2.75E-08	11	BTK, IGHA1, IGHA2, IGHD, IGHG2, IGHG3, IGHG4, IGHV3-49, IGKC, IGLL5, PNP
GO:0032989	cellular component morphogenesis	3.24E-05	2.89E-08	17	Actb, ACTN1, ALDOA, CALR, CAP1, CD9, CFL1, CORO1C, FERMT3, FLNA, ILK, ITGB3, Msn, MYH9, TPM1, VCL, WDR1
GO:0050865	regulation of cell activation	4.22E-05	3.76E-08	13	BTK, CD9, IGHA1, IGHA2, IGHD, IGHG2, IGHG3, IGHG4, IGHV3-49, IGKC, IGLL5, PLEK, PNP
GO:0002768	immune response-regulating cell surface receptor signaling pathway	4.70E-05	4.19E-08	12	Actb, ARPC1B, BTK, IGHA1, IGHA2, IGHD, IGHG2, IGHG3, IGHG4, IGHV3-49, IGKC, IGLL5
GO:0000902	cell morphogenesis	5.62E-05	5.01E-08	16	Actb, ACTN1, ALDOA, CALR, CAP1, CFL1, CORO1C, FERMT3, FLNA, ILK, ITGB3, Msn, MYH9, TPM1, VCL, WDR1
GO:0042113	B cell activation	6.01E-05	5.36E-08	10	BTK, IGHA1, IGHA2, IGHD, IGHG2, IGHG3, IGHG4, IGHV3-49, IGKC, IGLL5
GO:0002757	immune response-activating signal transduction	6.17E-05	5.50E-08	13	Actb, ARPC1B, BTK, HSPA1B, IGHA1, IGHA2, IGHD, IGHG2, IGHG3, IGHG4, IGHV3-49, IGKC, IGLL5
GO:0050851	antigen receptor-mediated signaling pathway	6.18E-05	5.51E-08	10	BTK, IGHA1, IGHA2, IGHD, IGHG2, IGHG3, IGHG4, IGHV3-49, IGKC, IGLL5
GO:0031589	cell-substrate adhesion	8.36E-05	7.45E-08	10	ACTN1, CALR, CORO1C, FERMT3, FLNA, ILK, ITGA2B, ITGB3, RSU1, VCL
GO:0050896	response to stimulus	8.94E-05	7.97E-08	48	Actb, ALDOA, ARHGDIB, ARPC1B, BTK, CALR, CAP1, CD9, CFL1, CORO1C, F13A1, FERMT3, FLNA, GAPDH, GP5, HSPA1B, IGFALS, IGHA1, IGHA2, IGHD, IGHG2, IGHG3, IGHG4, IGHV3-49, IGKC, IGKV3D-11, IGKV3D-7, IGLL5, ILK, ITGA2B, ITGB3, Msn, MYH9, MYL12A, PDIA3, PFN1, PKM, PLEK, PNP, PPIA, PRDX6, RAP1B, RSU1, TLN1, TMSB4X, TPM1, VCL, YWHAZ
GO:0032956	regulation of actin cytoskeleton organization	0.000108913	9.71E-08	10	ARHGDIB, ARPC1B, CFL1, FLNA, ILK, PFN1, PLEK, TMSB4X, TPM1, WDR1
GO:0002764	immune response-regulating signaling pathway	0.000110963	9.89E-08	13	Actb, ARPC1B, BTK, HSPA1B, IGHA1, IGHA2, IGHD, IGHG2, IGHG3, IGHG4, IGHV3-49, IGKC, IGLL5
GO:0006959	humoral immune response	0.000151863	1.35E-07	10	GAPDH, IGHA1, IGHA2, IGHD, IGHG2, IGHG3, IGHG4, IGHV3-49, IGKC, IGLL5
GO:0098609	cell-cell adhesion	0.000164914	1.47E-07	14	Actb, CD9, FERMT3, FLNA, ILK, ITGA2B, ITGB3, Msn, MYH9, MYL12A, PLEK, PNP, TLN1, VCL
GO:0051270	regulation of cellular component movement	0.000201194	1.79E-07	15	ACTN1, ARHGDIB, CALR, CD9, CORO1C, FERMT3, FLNA, ILK, ITGA2B, ITGB3, Msn, PFN1, TMSB4X, TPM1, VCL
GO:0030334	regulation of cell migration	0.000201506	1.80E-07	14	ARHGDIB, CALR, CD9, CORO1C, FERMT3, FLNA, ILK, ITGA2B, ITGB3, Msn, PFN1, TMSB4X, TPM1, VCL
GO:0002449	lymphocyte mediated immunity	0.000219393	1.96E-07	10	BTK, IGHA1, IGHA2, IGHD, IGHG2, IGHG3, IGHG4, IGHV3-49, IGKC, IGLL5
GO:0002460	adaptive immune response based on somatic recombination of immune receptors built from immunoglobulin superfamily domains	0.000224713	2.00E-07	10	BTK, IGHA1, IGHA2, IGHD, IGHG2, IGHG3, IGHG4, IGHV3-49, IGKC, IGLL5
GO:0002253	activation of immune response	0.000240794	2.15E-07	13	Actb, ARPC1B, BTK, HSPA1B, IGHA1, IGHA2, IGHD, IGHG2, IGHG3, IGHG4, IGHV3-49, IGKC, IGLL5
GO:1902905	positive regulation of supramolecular fiber organization	0.000255738	2.28E-07	8	ARPC1B, CFL1, FLNA, HSPA1B, PFN1, PLEK, TPM1, WDR1
GO:0050900	leukocyte migration	0.000266257	2.37E-07	11	CALR, CD9, IGHA1, IGHA2, IGKC, ITGA2B, ITGB3, Msn, MYH9, PPIA, WDR1
GO:0001667	ameboidal-type cell migration	0.000271522	2.42E-07	10	ARHGDIB, CALR, CAP1, CFL1, CORO1C, ILK, ITGB3, MYH9, PFN1, TMSB4X
GO:0048518	positive regulation of biological process	0.000274261	2.44E-07	38	Actb, ACTN1, ARPC1B, BTK, CALR, CFL1, CORO1C, FERMT3, FLNA, GAPDH, HSPA1B, IGHA1, IGHA2, IGHD, IGHG2, IGHG3, IGHG4, IGHV3-49, IGKC, IGLL5, ILK, ITGA2B, ITGB3, Msn, MYH9, PDIA3, PFN1, PKM, PLEK, PNP, PPIA, PRDX6, RAP1B, RSU1, TMSB4X, TPM1, WDR1, YWHAZ
GO:0046649	lymphocyte activation	0.000322044	2.87E-07	13	BTK, IGHA1, IGHA2, IGHD, IGHG2, IGHG3, IGHG4, IGHV3-49, IGKC, IGLL5, Msn, MYH9, PNP
GO:0051249	regulation of lymphocyte activation	0.000357368	3.19E-07	11	BTK, IGHA1, IGHA2, IGHD, IGHG2, IGHG3, IGHG4, IGHV3-49, IGKC, IGLL5, PNP
GO:0007163	establishment or maintenance of cell polarity	0.000375452	3.35E-07	8	Actb, CAP1, CFL1, ILK, Msn, MYH9, RAP1B, WDR1
GO:0002684	positive regulation of immune system process	0.000388774	3.47E-07	16	Actb, ARPC1B, BTK, CALR, HSPA1B, IGHA1, IGHA2, IGHD, IGHG2, IGHG3, IGHG4, IGHV3-49, IGKC, IGLL5, ITGA2B, PNP
GO:2000145	regulation of cell motility	0.000468498	4.18E-07	14	ARHGDIB, CALR, CD9, CORO1C, FERMT3, FLNA, ILK, ITGA2B, ITGB3, Msn, PFN1, TMSB4X, TPM1, VCL
GO:0048522	positive regulation of cellular process	0.000506915	4.52E-07	35	ACTN1, ARPC1B, BTK, CALR, CFL1, CORO1C, FERMT3, FLNA, GAPDH, HSPA1B, IGHA1, IGHA2, IGHD, IGHG2, IGHG3, IGHG4, IGHV3-49, IGKC, IGLL5, ILK, ITGA2B, ITGB3, Msn, MYH9, PDIA3, PFN1, PLEK, PNP, PPIA, PRDX6, RAP1B, TMSB4X, TPM1, WDR1, YWHAZ
GO:0034446	substrate adhesion-dependent cell spreading	0.000533577	4.76E-07	6	CALR, CORO1C, FERMT3, FLNA, ILK, ITGB3
GO:0051495	positive regulation of cytoskeleton organization	0.000540322	4.82E-07	8	ARPC1B, CFL1, FLNA, HSPA1B, PFN1, PLEK, TPM1, WDR1
GO:0002444	myeloid leukocyte mediated immunity	0.000697289	6.21E-07	11	ALDOA, BTK, CAP1, HSPA1B, PKM, PNP, PPIA, PRDX6, RAP1B, VCL, WDR1
GO:0051493	regulation of cytoskeleton organization	0.000762116	6.79E-07	11	ARHGDIB, ARPC1B, CFL1, FLNA, HSPA1B, ILK, PFN1, PLEK, TMSB4X, TPM1, WDR1
GO:0033622	integrin activation	0.000830428	7.40E-07	4	FERMT3, PLEK, RAP1B, TLN1
GO:0042742	defense response to bacterium	0.000896433	7.99E-07	9	IGHA1, IGHA2, IGHD, IGHG2, IGHG3, IGHG4, IGHV3-49, IGKC, IGLL5

Number of downregulated proteins equal to 59
The result table shows 59 downregulated proteins: 4 genes unannotated: No-name, calm1a, IGKV3D-11, and IGKV3D-7 ; 2 unkown: No-name and calm1a.
p-value <= 0.001

Supplementary Table S4 -Result table of gene ontology analysis for downregulated proteins considering the molecular function as classificator.

GO ID	Term	P-value	Uncorrected P-value	Number annotated	Annotated genes
GO:0003779	actin binding	6.82E-13	5.78E-15	17	ACTN1, ALDOA, ARPC1B, CAP1, CFL1, CORO1C, FLNA, Msn, MYH9, PFN1, TLN1, TMSB4X, TPM1, TPM3, TPM4, VCL, WDR1
GO:0050839	cell adhesion molecule binding	6.08E-12	5.15E-14	17	ACTN1, ALDOA, CALR, CD9, FERMT3, FLNA, ILK, ITGB3, Msn, MYH9, PFN1, PKM, PRDX6, TAGLN2, TLN1, VCL, YWHAZ
GO:0034987	immunoglobulin receptor binding	3.55E-11	3.01E-13	10	FLNA, IGHA1, IGHA2, IGHD, IGHG2, IGHG3, IGHG4, IGHV3-49, IGKC, IGLL5
GO:0051015	actin filament binding	1.75E-09	1.49E-11	11	ACTN1, ARPC1B, CFL1, CORO1C, FLNA, MYH9, TLN1, TPM1, TPM3, TPM4, WDR1
GO:0008092	cytoskeletal protein binding	4.47E-09	3.79E-11	19	Actb, ACTN1, ALDOA, ARPC1B, CAP1, CFL1, CORO1C, FLNA, GAPDH, Msn, MYH9, PFN1, TLN1, TMSB4X, TPM1, TPM3, TPM4, VCL, WDR1
GO:0044877	protein-containing complex binding	1.61E-08	1.36E-10	20	Actb, ACTN1, ARPC1B, CALR, CD9, CFL1, CORO1C, FERMT3, FLNA, ILK, ITGA2B, ITGB3, MYH9, PKM, RAP1B, TLN1, TPM1, TPM3, TPM4, WDR1
GO:0003823	antigen binding	5.63E-07	4.77E-09	9	IGHA1, IGHA2, IGHD, IGHG2, IGHG3, IGHG4, IGHV3-49, IGKC, IGLL5
GO:0005102	signaling receptor binding	7.19E-07	6.09E-09	21	ACTN1, CALR, CD9, CFL1, FERMT3, FLNA, HSPA1B, IGHA1, IGHA2, IGHD, IGHG2, IGHG3, IGHG4, IGHV3-49, IGKC, IGLL5, ILK, ITGB3, Msn, MYH9, TLN1
GO:0005178	integrin binding	9.23E-07	7.82E-09	8	ACTN1, CALR, CD9, FERMT3, ILK, ITGB3, MYH9, TLN1
GO:0005200	structural constituent of cytoskeleton	3.67E-06	3.11E-08	7	Actb, ARPC1B, Msn, TLN1, TPM1, TUBA1C, TUBA4A
GO:0045296	cadherin binding	4.23E-06	3.59E-08	10	ALDOA, FLNA, MYH9, PFN1, PKM, PRDX6, TAGLN2, TLN1, VCL, YWHAZ
GO:0005515	protein binding	1.37E-05	1.16E-07	54	Actb, ACTN1, ALDOA, ARHGDIB, ARPC1B, BTK, CALR, CAP1, CD9, CFL1, CORO1C, CP, FERMT3, FLNA, GAPDH, GP5, HSPA1B, IGFALS, IGHA1, IGHA2, IGHD, IGHG2, IGHG3, IGHG4, IGHV3-49, IGKC, IGLL5, ILK, ITGA2B, ITGB3, Msn, MYH9, MYL12A, PDIA3, PFN1, PKM, PLEK, PNP, PPIA, PRDX6, RAP1B, RSU1, SDPR, TAGLN2, TLN1, TMSB4X, TPM1, TPM3, TPM4, TUBA1C, TUBA4A, VCL, WDR1, YWHAZ

Number of downregulated proteins equal to 59
The result table shows 59 downregulated proteins: 4 genes unannotated: No-name, calm1a, IGKV3D-11, and IGKV3D-7 ; 2 unkown: No-name and calm1a.
p-value <= 0.001

Supplementary Table S5 - Result table of gene ontology analysis for upregulated proteins considering the cellular component as classificator.

GO ID	Term	P-value	Uncorrected P-value	Number annotated	Annotated genes
GO:0005615	extracellular space	1.83E-07	3.33E-09	11	HBB, HBG2, HGFAC, LAMP2, MASP2, MST1, PI16, PRDX2, SHBG, STXBP2, TNXB
GO:0044421	extracellular region part	3.61E-07	6.56E-09	11	HBB, HBG2, HGFAC, LAMP2, MASP2, MST1, PI16, PRDX2, SHBG, STXBP2, TNXB
GO:0005576	extracellular region	3.66E-06	6.66E-08	11	HBB, HBG2, HGFAC, LAMP2, MASP2, MST1, PI16, PRDX2, SHBG, STXBP2, TNXB

GO:0031838	haptoglobin-hemoglobin complex	0.000701262	1.28E-05	2	HBB, HBG2
GO:0005833	hemoglobin complex	0.000856837	1.56E-05	2	HBB, HBG2
GO:0070062	extracellular exosome	0.00190081	3.46E-05	7	HBB, LAMP2, MASP2, PRDX2, SHBG, STXBP2, TNXB
GO:1903561	extracellular vesicle	0.00202999	3.69E-05	7	HBB, LAMP2, MASP2, PRDX2, SHBG, STXBP2, TNXB
GO:0043230	extracellular organelle	0.00206181	3.75E-05	7	HBB, LAMP2, MASP2, PRDX2, SHBG, STXBP2, TNXB
GO:0070820	tertiary granule	0.00490937	8.93E-05	3	HBB, LAMP2, STXBP

Number of upregulated proteins equal to 11
The result table shows 11 genes annotated
p-value <=0.01

Supplementary Table S6 -Result table of gene ontology analysis for upregulated proteins considering the biological process as classificator.

GO ID	Term	P-value	Uncorrected P-value	Number annotated	Annotated genes
GO:0042744	hydrogen peroxide catabolic process	0.000115596	6.39E-07	3	HBB, HBG2, PRDX2
GO:0042743	hydrogen peroxide metabolic process	0.00064132	3.54E-06	3	HBB, HBG2, PRDX2
GO:0017001	antibiotic catabolic process	0.000713437	3.94E-06	3	HBB, HBG2, PRDX2
GO:0051187	cofactor catabolic process	0.00105546	5.83E-06	3	HBB, HBG2, PRDX2
GO:0098869	cellular oxidant detoxification	0.00380292	2.10E-05	3	HBB, HBG2, PRDX2
GO:0015671	oxygen transport	0.00466117	2.58E-05	2	HBB, HBG2
GO:1990748	cellular detoxification	0.00518364	2.86E-05	3	HBB, HBG2, PRDX2
GO:0098754	detoxification	0.00846904	4.68E-05	3	HBB, HBG2, PRDX2
GO:0015669	gas transport	0.00874556	4.83E-05	2	HBB, HBG2

Number of upregulated proteins equal to 11
The result table shows 11 genes annotated
p-value <=0.01

Supplementary Table S7 -Result table of gene ontology analysis for upregulated proteins considering the molecular function as classificator.

GO ID	Term	P-value	Uncorrected P-value	Number annotated	Annotated genes
GO:0004601	peroxidase activity	9.62E-05	2.83E-06	3	HBB, HBG2, PRDX2
GO:0031721	hemoglobin alpha binding	9.65E-05	2.84E-06	2	HBB, HBG2
GO:0016684	oxidoreductase activity, acting on peroxide as acceptor	0.000120469	3.54E-06	3	HBB, HBG2, PRDX2
GO:0030492	hemoglobin binding	0.000346912	1.02E-05	2	HBB, HBG2
GO:0031720	haptoglobin binding	0.000346912	1.02E-05	2	HBB, HBG2
GO:0016209	antioxidant activity	0.000440705	1.30E-05	3	HBB, HBG2, PRDX2
GO:0005344	oxygen carrier activity	0.000750726	2.21E-05	2	HBB, HBG2
GO:0004252	serine-type endopeptidase activity	0.00303488	8.93E-05	3	HGFAC, MASP2, MST1
GO:0008236	serine-type peptidase activity	0.00440731	0.000129627	3	HGFAC, MASP2, MST1
GO:0017171	serine hydrolase activity	0.00469367	0.000138049	3	HGFAC, MASP2, MST1
GO:0019825	oxygen binding	0.0056884	0.000167306	2	HBB, HBG2

Number of upregulated proteins equal to 11
The result table shows 11 genes annotated
p-value <=0.01

-Supplementary Table S8-

Table S8. Summary of mutations in *ATP7A* gene

Exon	Nucleotide	Location in protein domains (ATP7A)	Phenotype	Population/ number of cases	References
3	c.611_612AA del	Near the MBD2	MD		
4	c.818_819ATdel	Between the MBD2 and MBD3	MD		
4	c.979Adel	Near the MBD3	MD		
4	c.1168Gdel	Between the MBD3 and MBD4	MD		
9	c.2157_2158ATin	TM1	MD		
10	c.2499_2500Cins	TM4	MD		
18	c.3778Gdel	Between the P-domain and N-domain	MD		
18	c.3802Tdel	Between the P-domain and N-domain	MD	Japanese / 66	[1]
4	c.1152A>T	Between the MBD3 and MBD4	MD		
4	c.1175A>G	Between the MBD3 and MBD4	MD		
13	c.2828A>T	Near the A-domain	MD		
15	c.3102G>A	TM6	MD		
16	c.3282C>T	P-domain	MD		
20	c.4060C>G	N-domain	MD		
23	c.A4394G	Near the TM8	MD		
4	c.1403G>T	Near the MBD4	MD		
5	c.1618C>A	Near the MBD5	MD		
6	c.1793G>T	Between the MBD5 and MBD6	MD		
15	c.2972C>A	TM6	dHMN	Italian / 2	[2]
21	c.4067G>C	TM7	MD	American / 1	[3]
22	c.4222A > T	TM8	OHS	Italian / 1	[4]
4	c.907C > T	MBD3	MD		
5	c.1519G > T	MBD5	MD		
7	c.1747G > T	MBD6	MD		
15	c.3067_3096del30	P-domain	MD		
21	c.4108_4113delATTCCC	TM7	MD		
5	c.1355_1356delTA	MBD4	MD	Italian / 25	[5]
3	c.467delA	MBD1	MD		
4	c.865_868delAAATins24	MBD3	MD		
10	c.2313dupG	Between TM3 and TM4	MD		
18	c.3539 T > A	N-domain	MD		

22	c.4222A > T	TM8	MD		
-	c.2179G > A	-	MD	Chinese / 24	[6]
-	c.3352G>A	N-domain	MD	Japanese / 11	[7]
3	c.532G>T	MBD2	MD	European / 517	[8]
3	c.589C>T	MBD2	MD	American / 24	[9]
3	c.601C>T	MBD2	MD	American / 1	[10]
5	c.1460C>A	MBD5	MD	Japanese / 17	[11]
6	c.1585G>T	MBD5	MD	Japanese / 35	[12]
6	c.1639C>T	MBD5	MD	European / 341	[13]
7	c.1734C>A	MBD6	MD	American / 41	[14]
8	c.1874T>R	MBD6	MD	American / 24	[9]
12	c.2531G>A	A-domain	MD		
12	c.2579G>T	A-domain	MD		
12	c.2618T>G	A-domain	MD		
12	c.2626G>C	A-domain	MD	*	[15]
13	c.2771G>A	A-domain	MD		
15	c.2998T>C	TM6	MD		
16	c.3131A>G	P-domain	MD		
20	c.3844A>G	P-domain	MD		
20	c.3974C>T	P-domain	MD	European / 2	[16]
15	c.3043G>A	TM6	MD		
8	c.1885G>C	MBD1	MD		
9	c.2059A>G	TM1	MD		
10	c.2179G>A	TM1	MD		
12	c.2609A>G	P-domain	MD		
15	c.3013G>A	N-domain	MD	European / 40	[17]
15	c.3043G>A	N-domain	MD		
16	c.3143C>T	P-domain	MD		
16	c.3293A>C	P-domain	MD		
22	c.4177A>G	TM8	MD		
4	c.721C>T	MBD2	MD		
15	c.2944G>T	N-domain	MD		
21	c.4085C>A	TM7	MD	*	[15]
22	c.4117G>C	TM7	MD	*	[18]
22	c.4118C>T	TM7	MD		
22	c.4190C>T	TM8	MD	American / 36	[19]
22	c.4190C>T	TM8	MD	*	[15, 20]

Menkes disease (MD); Occipital horn syndrome (OHS); Distal hereditary motor neuropathy (dHMN); Del: deletion; In: insertion; MBD: heavy metal binding domain (Cu, 1-6); TM: transmembrane domain (1-8); A domain: activation domain; P domain: phosphorylation domain and; N domain: nucleotide domain. *Non reported

References

1. Fujisawa, C., et al., *ATP7A mutations in 66 Japanese patients with Menkes disease and carrier detection: A gene analysis.* Pediatr Int, 2019. **61**(4): p. 345-350.
2. Gualandi, F., et al., *Report of a novel ATP7A mutation causing distal motor neuropathy.* Neuromuscul Disord, 2019. **29**(10): p. 776-785.
3. Caicedo-Herrera, G., et al., *Novel ATP7A gene mutation in a patient with Menkes disease.* Appl Clin Genet, 2018. **11**: p. 151-155.
4. Bonati, M.T., et al., *A novel nonsense ATP7A pathogenic variant in a family exhibiting a variable occipital horn syndrome phenotype.* Mol Genet Metab Rep, 2017. **13**: p. 14-17.
5. de Gemmis, P., et al., *13 novel putative mutations in ATP7A found in a cohort of 25 Italian families.* Metab Brain Dis, 2017. **32**(4): p. 1173-1183.
6. Cao, B., et al., *Identification of novel ATP7A mutations and prenatal diagnosis in Chinese patients with Menkes disease.* Metab Brain Dis, 2017. **32**(4): p. 1123-1131.
7. Kim, J.H., et al., *Novel mutations and clinical outcomes of copper-histidine therapy in Menkes disease patients.* Metab Brain Dis, 2015. **30**(1): p. 75-81.
8. Moller, L.B., et al., *Clinical expression of Menkes disease in females with normal karyotype.* Orphanet J Rare Dis, 2012. **7**: p. 6.
9. Kaler, S.G., et al., *Molecular correlates of epilepsy in early diagnosed and treated Menkes disease.* J Inherit Metab Dis, 2010. **33**(5): p. 583-9.
10. Kaler, S.G., et al., *Translational read-through of a nonsense mutation in ATP7A impacts treatment outcome in Menkes disease.* Ann Neurol, 2009. **65**(1): p. 108-13.
11. Gu, Y.H., et al., *ATP7A gene mutations in 16 patients with Menkes disease and a patient with occipital horn syndrome.* Am J Med Genet, 2001. **99**(3): p. 217-22.
12. Gu, Y.H., H. Kodama, and T. Kato, *Congenital abnormalities in Japanese patients with Menkes disease.* Brain Dev, 2012. **34**(9): p. 746-9.
13. Tumer, Z., *An overview and update of ATP7A mutations leading to Menkes disease and occipital horn syndrome.* Hum Mutat, 2013. **34**(3): p. 417-29.
14. Tumer, Z., et al., *Identification of point mutations in 41 unrelated patients affected with Menkes disease.* Am J Hum Genet, 1997. **60**(1): p. 63-71.
15. Moller, L.B., et al., *Identification and analysis of 21 novel disease-causing amino acid substitutions in the conserved part of ATP7A.* Hum Mutat, 2005. **26**(2): p. 84-93.
16. Borm, B., et al., *Variable clinical expression of an identical mutation in the ATP7A gene for Menkes disease/occipital horn syndrome in three affected males in a single family.* J Pediatr, 2004. **145**(1): p. 119-21.
17. Moizard, M.P., et al., *Twenty-five novel mutations including duplications in the ATP7A gene.* Clin Genet, 2011. **79**(3): p. 243-53.
18. Gourdon, P., et al., *Crystal structure of a copper-transporting PIB-type ATPase.* Nature, 2011. **475**(7354): p. 59-64.
19. Skjorringe, T., et al., *Characterization of ATP7A missense mutants suggests a correlation between intracellular trafficking and severity of Menkes disease.* Sci Rep, 2017. **7**(1): p. 757.
20. de Bie, P., et al., *Molecular pathogenesis of Wilson and Menkes disease: correlation of mutations with molecular defects and disease phenotypes.* J Med Genet, 2007. **44**(11): p. 673-88.
